# Robust Stackelberg-Supervised Overtaking Policy with Persistent Tactical Corridors for Autonomous Racing

**DOI:** 10.3390/s26217036

**Published:** 2026-11-06

**Authors:** Shuyi Gao, Ren Jin, Defu Lin, Weiqiang Xiao, Jinmeng Chen

**Affiliations:** School of Aerospace Engineering, Beijing Institute of Technology, Beijing 100081, China; 3120235037@bit.edu.cn (S.G.); lindf@bit.edu.cn (D.L.); 3220230018@bit.edu.cn (W.X.); 1120230188@bit.edu.cn (J.C.)

**Keywords:** autonomous racing, game-guided reinforcement learning, tactical corridor, overtaking policy, sensor-based decision-making

## Abstract

Overtaking planning and decision-making are central to performance in autonomous racing. Limited maneuvering space on narrow closed circuits makes high-speed overtaking difficult and increases the risk of failed overtaking attempts and collisions. To address this problem, we formulate overtaking as robust tactical feasible-set selection and develop a Stackelberg-supervised policy that translates response-set reasoning into persistent tactical corridors for constrained planning and closed-loop control. First, a robust Stackelberg response-set teacher provides action-prior and action-conditioned robust-value supervision to a Truncated Quantile Critics (TQC) policy that takes sensor-derived opponent-state estimates as input and learns passing-side intention, corridor parameters, and speed demand. Second, a hysteretic finite-state machine (FSM) preserves the selected passing side, while a deterministic corridor carver combines the learned corridor parameters with the evolving track and interaction geometry to construct a time-varying Frenet corridor. Finally, a corridor-constrained sequential second-order cone programming (SOCP) planner and a speed profiler generate a continuous path–speed reference, which is tracked using reference-conditioned contouring-error nonlinear model predictive control (RC-NMPC) to produce closed-loop vehicle commands. Hardware-in-the-loop experiments were conducted using an Emirates Autonomous Vehicle (EAV24) formula race-car simulator with Gaussian noise and packet drops applied to localization and perception outputs. Across 1000 laps, the method achieved a lead-acquisition rate of 94.7% and a mean time to lead acquisition of 4.82 s. Compared with three baselines using the same execution stack, the lead-acquisition rate was 31.2–66.2 percentage points higher and the mean time to lead acquisition was 3.01–6.41 s shorter; no collisions or track-boundary violations were observed.

## 1. Introduction

Autonomous racing provides a demanding testbed for integrated perception, decision-making, planning, and control under extreme operating conditions. Competitions such as the Indy Autonomous Challenge and the Abu Dhabi Autonomous Racing League (A2RL) have extended this testbed to full-scale head-to-head racing near the limits of vehicle handling [1,2]. In these races, the ability to complete overtaking maneuvers safely and efficiently directly affects competitive performance. Overtaking is especially difficult on narrow closed circuits, where high speed and limited lateral space shorten the passing window and increase collision risk. Onboard localization and opponent perception provide the evolving vehicle-state estimates on which these overtaking decisions are based. A viable overtaking decision must retain its passing-side intent across repeated replanning and leave sufficient maneuvering freedom for downstream path generation and vehicle control. The central challenge is therefore to translate interactive tactical reasoning into a geometrically explicit and temporally persistent feasible region throughout the overtaking maneuver.

Integrated racing systems combine sensor-based localization and environmental perception with planning and control [3,4]. Full-scale platforms use GNSS/IMU measurements for ego-state estimation and multi-sensor detection and tracking for opponent-state estimation [2,5]. Beyond instantaneous state estimates, Baumann et al. use Gaussian-process models to infer opponent trajectories from noisy LiDAR observations and guide overtaking [6]. Toschi et al. translate predicted opponent occupancies into drivable Frenet-frame tunnels that constrain model predictive control [7]. These approaches link sensor measurements to collision-aware planning through complementary representations of vehicle states, future opponent motion and available driving space.

Research on overtaking in autonomous racing has developed along four main directions: hierarchical maneuver planning, explicit game-theoretic planning, learning-based decision-making, and hybrid learning–optimization architectures. In hierarchical architectures, a tactical layer selects among following, defending, and passing behaviors, while a lower layer converts the selected behavior into a path and speed profile. Finite-state and candidate-trajectory planners provide a structured basis for real-time computation and allow racing rules, collision checks, and vehicle constraints to be handled by dedicated modules [8,9]. This modular structure is also adopted in full-scale head-to-head racing systems, where opponent prediction and local planning operate under tight real-time constraints [2,3]. Within such a hierarchy, Thakkar et al. formulated the high-level decision as a discrete racing game that generates target waypoints for either a multi-agent reinforcement learning controller or a linear-quadratic Nash-game controller [10]. These approaches provide interpretable tactical logic and a practical route to real-time implementation. Their tactical outputs, however, are commonly represented by maneuver states, target waypoints, or selected trajectories and provide only limited continuous parameterization of the spatial extent available to downstream planning.

Explicit game-theoretic planners model the coupling between competitors more directly. Non-cooperative dynamic games jointly evaluate progress, collision avoidance, blocking, and overtaking, producing behaviors such as shielding, faking, and opportunistic passing that are difficult to obtain from independent trajectory optimization [11,12]. Iterative linear-quadratic approximations reduce the computational cost of solving nonlinear multi-player games, although their local solution quality remains sensitive to the interaction model and initialization [13]. Hierarchical Stackelberg formulations separate the leader’s maneuver choice from the follower’s response, while payoff learning adapts the interaction objective from observed behavior [14,15]. Level-*K* reasoning offers a bounded model of opponent rationality; trajectory mixing can further hedge against changes in the inferred reasoning level [16]. Constrained-potential and near-potential formulations instead exploit common structure in the players’ objectives to reduce the online game-solving burden [17,18]. Collectively, these methods make strategic coupling explicit. Despite computational approximations, repeatedly solving the game online remains demanding under the real-time constraints of autonomous racing.

Learning-based methods replace repeated online search with fixed-cost policy inference and acquire interaction patterns directly from competitive experience. Curriculum learning has produced overtaking policies by gradually increasing opponent difficulty, while large-scale deep reinforcement learning has demonstrated high-performance racing and human-like tactical behavior in simulation [19,20,21]. Comparisons with optimal control show that reinforcement learning can exploit complex racing dynamics effectively, although its behavior depends strongly on the training distribution and reward design [22]. Other learning-based designs condition policies on nominal trajectories or infer actions from local LiDAR observations [23,24]. Data-driven predictive planners use learned opponent forecasts to generate overtaking trajectories [6]. Game-informed deep reinforcement learning couples the policy reward to another vehicle’s action, whereas dynamic-graph and multi-agent formulations encode pairwise relations or coordinated behavior among several vehicles [25,26,27]. Human feedback provides another source of policy guidance for stabilizing learned driving strategies [28]. These methods improve interaction awareness. Direct-control policies and single-trajectory outputs, however, leave limited freedom for constraint-aware replanning as the interaction evolves during overtaking.

Hybrid learning–optimization architectures therefore expose an intermediate decision to a model-based planner. Reiter et al. used reinforcement learning to parameterize the objective of a nonlinear predictive planner, confining exploration to trajectories accepted by the optimizer [29]. In road-traffic applications, learned tactical layers have been used to anticipate disruptive lane changes and to coordinate merging sequences subsequently realized by optimal control [30,31]. Most closely related to corridor-based planner interfaces, Yang et al. used MARL-generated cooperative reference trajectories to construct spatio-temporal corridors for downstream optimization [32]. Toschi et al. constructed alternative drivable tunnels from the predicted occupancies of other vehicles, selected a tunnel according to the current interaction, and optimized a trajectory within it [7]. Across these approaches, intermediate outputs parameterize the planning objective, provide a guiding trajectory, or select among separately generated corridors; the corridor parameters themselves are not learned as tactical outputs.

In summary, despite substantial progress, maintaining a consistent overtaking intent while preserving sufficient geometric freedom for constrained replanning remains challenging throughout a high-speed overtaking maneuver. This motivates a decision framework that represents this intent as a persistent tactical feasible region and communicates game-supervised tactical decisions to the planner through side-locked Frenet bounds and speed guidance.

To address this challenge, this study formulates interactive overtaking as robust tactical feasible-set selection and develops a game-guided tactical-corridor decision framework. The framework converts robust Stackelberg response-set reasoning into a persistent tactical feasible region and speed guidance for constrained planning and closed-loop control. Specifically, the main contributions are as follows:

1. Robust Stackelberg Response-Set Supervision: We developed a robust Stackelberg response-set teacher that evaluates constraint-admissible tactical candidates against near-best follower responses. The teacher then derives a response-set-optimal action prior and action-conditioned robust-value labels, thereby supervising a Truncated Quantile Critics (TQC) actor–critic method to learn robust overtaking decisions.
2. Persistent Tactical Feasible-Region Interface: We introduce a persistent tactical feasible-region interface that converts TQC outputs into a side-locked, time-varying Frenet corridor with speed guidance. A hysteretic FSM and deterministic opponent-footprint carving adapt the feasible region to evolving track and interaction geometry, enabling continuous support for path planning via sequential second-order cone programming (SOCP) and reference-conditioned contouring-error nonlinear model predictive control (RC-NMPC) throughout overtaking. For sensor-based racing, it links GNSS/IMU and LiDAR estimates to persistent planning constraints.
3. System Validation under High-Speed Conditions: We deployed the complete decision–planning–control stack on the target onboard computer and evaluated it using an EAV24 hardware-in-the-loop platform under high-speed head-to-head racing conditions. Matched baseline comparisons and ablation studies were also conducted. The results demonstrate a high lead-acquisition rate and collision-free closed-loop operation, highlighting the framework’s potential for practical deployment in autonomous racing.

The rest of this article is organized as follows. Section 2 formulates the problem and vehicle–track model. Section 3 and Section 4 present the tactical-corridor framework and its planning–control realization, respectively. Section 5 reports the experimental results and discussion, and Section 6 concludes the paper.

## 2. Problem Description and Modeling

**Figure 1:**
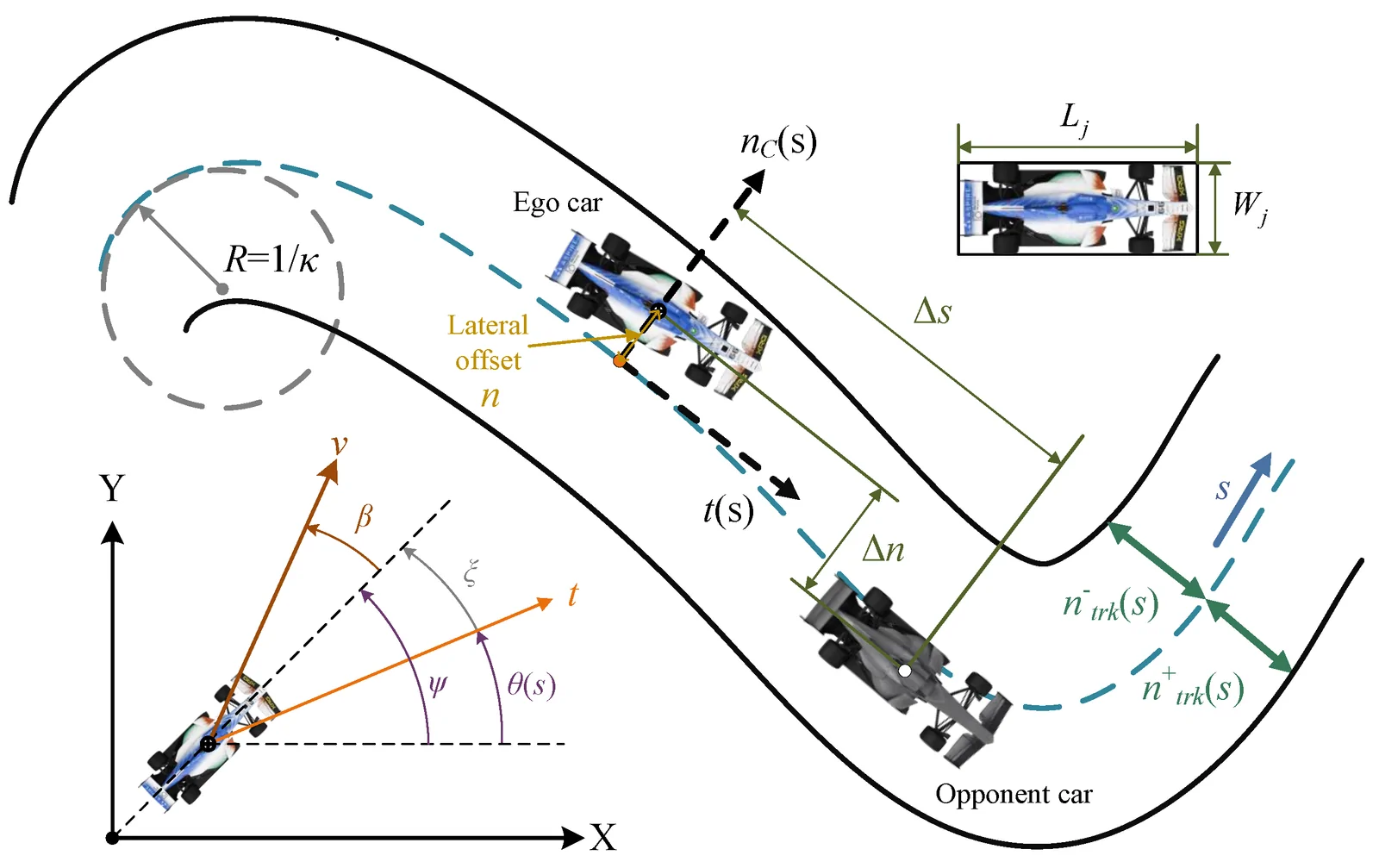
Vehicle motion and relative geometry in the Frenet frame.

Figure 1 shows the two-vehicle overtaking problem considered in this study, in which the ego race car must overtake a non-cooperative opponent on a closed track. The following models define the vehicle and interaction quantities used by the tactical policy and executor. Both vehicles are represented by identical rectangular footprints centered at their state reference points, with length $L_{j}=5.2$ m and width $W_{j}=1.9$ m, j∈{e,o}.

For vehicle i∈{e,o}, $\left(X_{i},Y_{i},\psi_{i}\right)$ denotes the inertial pose and $\left(v_{x,i},v_{y,i},r_{i}\right)$ the body-frame velocity and yaw rate, with $V_{i}=\sqrt{v_{x,i}^{2}+v_{y,i}^{2}}$ and $\beta_{i}=\mathrm{atan2}\left(v_{y,i},v_{x,i}\right)$. Let W={fl,fr,rl,rr}, and let $r_{q}={\left[x_{q},y_{q}\right]}^{⊤}$ and $\delta_{q}$ denote the body-frame position and steering angle of wheel *q*. Wheel-level quantities are vehicle specific, with the vehicle index omitted for clarity. The front wheels share the commanded steering angle and the rear wheels are unsteered. The planar four-wheel dynamics are given by Equation (1):

(1) $$\left[\begin{array}{c} {\dot{X}}_{i}\\ {\dot{Y}}_{i}\\ {\dot{\psi }}_{i}\\ {\dot{v}}_{x,i}\\ {\dot{v}}_{y,i}\\ {\dot{r}}_{i} \end{array}\right]=\left[\begin{array}{c} v_{x,i}\cos\psi_{i}-v_{y,i}\sin\psi_{i}\\ v_{x,i}\sin\psi_{i}+v_{y,i}\cos\psi_{i}\\ r_{i}\\ \begin{array}{c} \frac{1}{m}\sum_{q\in W}\left(F_{x,q}\cos\delta_{q}-F_{y,q}\sin\delta_{q}\right)+r_{i}v_{y,i}\\ -\frac{F_{\mathrm{drag}}\left(V_{i}\right)}{m} \end{array}\\ \frac{1}{m}\sum_{q\in W}\left(F_{x,q}\sin\delta_{q}+F_{y,q}\cos\delta_{q}\right)-r_{i}v_{x,i}\\ \begin{array}{c} \frac{1}{I_{z}}\sum_{q\in W}[x_{q}\left(F_{x,q}\sin\delta_{q}+F_{y,q}\cos\delta_{q}\right)\\ -y_{q}\left(F_{x,q}\cos\delta_{q}-F_{y,q}\sin\delta_{q}\right)] \end{array} \end{array}\right].$$

Here *m* and $I_{z}$ are the vehicle mass and yaw inertia, and $F_{\mathrm{drag}}$ is the aerodynamic drag force. The identified pure-slip tire forces are represented by Equation (2):

(2) $$\begin{array}{cc} F_{d,q}\left(\chi_{d,q},F_{z,q}\right) & =D_{d}\left(F_{z,q}\right)\sin\;\left(C_{d}\arctan\left(B_{d}\chi_{d,q}\right)\right),\\ D_{d}\left(F_{z,q}\right) & =F_{z,q}\;\left[\mu_{d}+s_{d}\frac{F_{z,q}-F_{z,0}}{F_{z,0}}\right],\\ B_{d} & =\frac{\tan\;\left(\pi /(2C_{d})\right)}{q_{d,\mathrm{pk}}}. \end{array}$$

Here $\chi_{x,q}=\lambda_{q}$ and $\chi_{y,q}=\alpha_{q}$. The resultant wheel forces determine the path-tangential and path-normal accelerations $a_{T}$ and $a_{N}$. Following [33], their admissible combinations are represented by the speed-interpolated envelope in Equation (3):

(3) $$A_{\mathrm{tire}}\left(V\right)=\left\{\left(a_{T},a_{N}\right):\Phi_{\mathrm{tire}}\left(a_{T},a_{N},V\right)\leq 1\right\}.$$

Here $\Phi_{\mathrm{tire}}$ is the normalized utilization returned by the interpolated map, with $\Phi_{\mathrm{tire}}=1$ defining the envelope boundary. The stationwise traction and braking magnitudes used in path planning are denoted by $a_{T,k}^{+}$ and $b_{T,k}^{-}$, respectively.

Let $r_{c}\left(s\right)$ denote the track centerline parameterized by arc length $s\in [0,L_{\mathrm{trk}})$, with tangent angle θ(s) and curvature κ(s)=dθ/ds, and let t(s) and $n_{c}\left(s\right)$ be its unit tangent and normal. With $p_{i}={\left[X_{i},Y_{i}\right]}^{⊤}$, the Frenet coordinates satisfy $p_{i}=r_{c}\left(s_{i}\right)+n_{i}n_{c}\left(s_{i}\right)$. Define the vehicle-body heading error as $\xi_{i}=\psi_{i}-\theta \left(s_{i}\right)$. The path-aligned velocity components $v_{s,i}=V_{i}\cos\left(\xi_{i}+\beta_{i}\right)$ and $v_{n,i}=V_{i}\sin\left(\xi_{i}+\beta_{i}\right)$ give Equation (4):

(4) $$\left[\begin{array}{c} {\dot{s}}_{i}\\ {\dot{n}}_{i}\\ {\dot{\xi }}_{i} \end{array}\right]=\left[\begin{array}{c} v_{s,i}/\left(1-\kappa \left(s_{i}\right)n_{i}\right)\\ v_{n,i}\\ r_{i}-\kappa \left(s_{i}\right)v_{s,i}/\left(1-\kappa \left(s_{i}\right)n_{i}\right) \end{array}\right].$$

The lateral coordinate is positive to the left, with $n_{\mathrm{trk}}^{-}\left(s\right)\leq n\leq n_{\mathrm{trk}}^{+}\left(s\right)$. Centerline projection gives the relative ego–opponent state in Equation (5):

(5) $$\Delta s={\mathrm{wrap}}_{L_{\mathrm{trk}}}\left(s_{o}-s_{e}\right),\;\Delta n=n_{o}-n_{e},\;\Delta v=v_{s,e}-v_{s,o}.$$

Here ${\mathrm{wrap}}_{L_{\mathrm{trk}}}\left(\cdot \right)$ returns the signed arc-length difference in $\left[-L_{\mathrm{trk}}/2,L_{\mathrm{trk}}/2\right)$; Δs>0 denotes an opponent ahead and Δv>0 denotes the ego closing. Under the adopted A2RL racing rule, the first sampled instant satisfying Δs<0 defines the Lead event. Side-lock release requires longitudinal separation of the two vehicle footprints:

(6) $$h_{s,j,t}=\frac{1}{2}\left(L_{j}|\cos\xi_{j,t}|+W_{j}\left|\sin\xi_{j,t}\right|\right),\;j\in \left\{e,o\right\},\;-\Delta s_{t}\geq g_{\mathrm{ot}}\geq h_{s,e,t}+h_{s,o,t}+d_{\mathrm{safe}}.$$

The side lock is released after Equation (6) holds for $N_{\mathrm{post}}$ consecutive tactical updates; $P_{t}=1$ denotes this confirmation.

For geometric path generation, let $\psi_{p}\left(s\right)$ denote the planned-path tangent angle and define $\xi_{p}\left(s\right)=\psi_{p}\left(s\right)-\theta \left(s\right)$. At the initial station, the path tangent is aligned with the ego velocity direction, giving $\xi_{p}\left(s_{e}\right)=\xi_{e}+\beta_{e}$. With *s* as the independent variable, $z\left(s\right)={\left[n\left(s\right),\xi_{p}\left(s\right)\right]}^{⊤}$ is the geometric state and $\omega \left(s\right)=d\psi_{p}/d\ell$ is the planned-path curvature, where *ℓ* is path arc length. The spatial model is given in Equation (7):

(7) $$z^{\prime }\left(s\right)=f_{s}\left(z,\omega ;s\right)=\left[\begin{array}{c} \left(1-\kappa n\right)\tan\xi_{p}\\ \left(1-\kappa n\right)\omega /\cos\xi_{p}-\kappa \end{array}\right].$$

## 3. Game-Supervised Tactical Feasible-Region Framework for Interactive Overtaking

The proposed tactical layer represents interactive overtaking decisions as persistent feasible regions for constrained motion planning. A robust Stackelberg response-set teacher evaluates constraint-admissible tactical candidates against near-best follower responses and generates a response-set-optimal action prior together with action-conditioned robust-value labels. A projected-action TQC actor–critic method distills this supervision into passing-side intention, corridor parameters, and speed demand. A hysteretic FSM preserves the selected passing side, while a deterministic opponent-footprint carver converts the learned corridor parameters and evolving interaction geometry into time-varying Frenet corridors.

### 3.1. Robust Stackelberg Response-Set Supervision

At each tactical update, the encoder combines the two vehicle states, local track preview, current mode, side lock, and execution health into ${\tilde{o}}_{t}$. The actor produces the action in Equation (8):

(8) $$a^{\mathrm{raw}}={\left[L^{\mathrm{cap}},R^{\mathrm{cap}},d_{\mathrm{safe}},V_{\mathrm{cap}}^{\mathrm{req}},g_{\mathrm{chase}},g_{\mathrm{ot}},g_{\mathrm{abort}},b_{\mathrm{lat}},w_{\mathrm{safe}},q_{\mathrm{side}}\right]}^{⊤}$$

where the components specify the left and right lateral caps, footprint clearance, requested speed cap, three mode-transition distances, defensive lateral offset and width, and passing-side intention. Together, they parameterize a planner-compatible tactical feasible region with speed guidance. The Euclidean projection $\Pi_{A}$ maps the raw action into the admissible parameter domain by enforcing the configured component bounds, the ordered transition thresholds $g_{\mathrm{ot}}+\Delta_{g}\leq g_{\mathrm{chase}}\leq g_{\mathrm{abort}}-\Delta_{g}$, and the minimum corridor width. This produces the bound-projected action $a^{B}$, which is subsequently processed by the tire-admissibility projection $\Pi_{\mathrm{tire}}$ to obtain the final tactical action $a^{F}$.

The offline teacher forms deterministic leader and follower banks by combining the three side codes with a Sobol design over the remaining action components. Every candidate is passed through the same bound projection, corridor carver, and tire screen used online; duplicate or infeasible candidates are discarded. Let $s_{t}^{G}=\left({\tilde{o}}_{t},\chi_{t}^{G}\right)$ denote the full rollout state available to the offline teacher, where $\chi_{t}^{G}$ collects the internal publisher, executor, and one-step reward memory required to evaluate a coupled candidate preview. The deployed policy is conditioned on the 51-dimensional observation ${\tilde{o}}_{t}$, with $\chi_{t}^{G}$ entering teacher-to-policy transfer as latent rollout context. Let $A_{i}^{G}\left(s_{t}^{G}\right)$ denote the candidate bank for role *i*. Each candidate pair induces the coupled executable preview in Equation (9):

(9) $$\left(R_{e},R_{o}\right)=P_{H_{G}}\;\left(s_{t}^{G};a_{e}^{F},a_{o}^{F}\right),$$

where $H_{G}$ denotes the number of tactical updates in the coupled offline teacher rollout. The operator $P_{H_{G}}$ recursively applies the stateful publisher in Algorithm 1 and the common execution stack. The candidate pair remains fixed over the preview while both vehicle and hybrid states evolve jointly; both utilities use the corresponding role-relative features of this preview.
**Algorithm 1** Game-supervised tactical-corridor policy.**Require:** teacher states and replay D; online states, track preview, previous $\left(m_{t-1},\sigma_{t-1}^{\mathrm{lock}},C_{t-1}\right)$, and $h_{t}$; last freshly validated corridor, speed cap and reuse mark (initially absent)  1:**Offline teacher and training:**  2:**for** each teacher state **do**  3:    combine Sobol samples with left/neutral/right codes; apply $a^{\mathrm{raw}}\;\to \;a^{B}\;\to \;a^{F}$  4:    discard duplicate or infeasible candidates; generate the coupled preview (9) for each candidate pair  5:    if either admissible bank is empty, skip this teacher state  6:    evaluate (10), retain near-best follower responses, and compute the robust teacher action (12)  7:    store every matched $\left(\tilde{o},a^{F},v_{g},a_{\mathrm{th}}^{⋆}\right)$ in $D_{g}$  8:**end for**  9:update TQC from D and the prior/value terms from $D_{g}$ using the combined TQC and teacher-supervision losses10:**Online tactical publication:**11:sample $a^{\mathrm{raw}}$, apply $\Pi_{A}$, predict the opponent at constant Frenet velocity, and evaluate the available side widths12:**if** $h_{t}$ is invalid, or a locked state satisfies $\Delta s_{t}\geq g_{\mathrm{abort}}$ **then**13:    set Hold and release the side lock; invalidate the stored corridor and return a braking-fallback request14:**else if** the locked ego satisfies the clearance-confirmation guard $P_{t}=1$ **then**15:    return to Raceline and release the side lock16:**else if** a lock exists, or a non-neutral feasible side is requested with $0<\Delta s_{t}\leq g_{\mathrm{chase}}$ and $\Delta v_{t}\geq 0$ **then**17:    retain/commit that side; select Shadow for $\Delta s_{t}>g_{\mathrm{ot}}$ and Overtake otherwise18:**else if** $\sigma^{\mathrm{lock}}=0$, $-g_{\mathrm{chase}}\leq \Delta s_{t}<0$, and $\Delta v_{t}<0$ **then**19:    select Defend20:**else**21:    select Raceline22:**end if**23:construct the mode interval using the opponent-footprint exclusion rule and the corresponding raceline, defend, or connected-hold rule; use η=1 in Overtake and the gap-based ramp in Shadow24:apply the tire-admissibility projection; if no trial cap is admissible, invoke current-state Hold and braking fallback; invalidate the stored corridor, release the lock, and return immediately25:apply immediate contraction and smoothed relaxation;26:**if** the new corridor satisfies $L_{\min}^{\mathrm{corr}}$ **then**27:    cache the new corridor and speed cap; clear the reuse mark28:**else if** an unused cached corridor and cap pass current-state checks for the selected side/mode **then**29:    restore the cached corridor and cap; mark used without refreshing the timestamp30:**else**31:    invalidate the cache, release the lock, and return a Hold/braking request32:**end if**33:**return** $G_{t}$ or a braking-fallback request

Let $Z_{g}=\sum_{k=0}^{H_{G}-1}\gamma_{g}^{k}$ and $c_{w}=\sum_{j=1}^{10}{\left[-w_{j}\right]}_{+}$, where ${\left[x\right]}_{+}=\max\left(x,0\right)$. The normalized role-relative utility is defined in Equation (10):

(10) $$U_{i}\left(s_{t}^{G},a_{e}^{F},a_{o}^{F}\right)=\frac{1}{Z_{g}{∥w∥}_{1}}\sum_{k=0}^{H_{G}-1}\gamma_{g}^{k}\left[w^{⊤}\phi_{i,k}\left(R_{e},R_{o}\right)+c_{w}\right],$$

where $\phi_{i}\in {\left[0,1\right]}^{10}$ contains the role-relative reward features in Appendix A. Hence $U_{i}\in \left[0,1\right]$, and $\varepsilon_{\mathrm{BR}}$ directly specifies the admissible follower-utility gap. For a fixed ego candidate, let $U_{o}^{\max}\left(s_{t}^{G},a_{e}^{F}\right)$ denote the largest follower utility over $A_{o}^{G}\left(s_{t}^{G}\right)$. The near-best response set is defined in Equation (11):

(11) $$R_{o}^{\varepsilon }\left(s_{t}^{G},a_{e}^{F}\right)=\left\{a_{o}^{F}\in A_{o}^{G}\left(s_{t}^{G}\right):\;U_{o}\left(s_{t}^{G},a_{e}^{F},a_{o}^{F}\right)\geq U_{o}^{\max}\left(s_{t}^{G},a_{e}^{F}\right)-\varepsilon_{\mathrm{BR}}\right\}.$$

The teacher evaluates each candidate against its least favorable retained response and selects the strongest robust action using Equation (12):

(12) $$\begin{array}{cc} V_{g}\left(s_{t}^{G},a_{e}^{F}\right) & =\min_{a_{o}^{F}\in R_{o}^{\varepsilon }\left(s_{t}^{G},a_{e}^{F}\right)}U_{e}\left(s_{t}^{G},a_{e}^{F},a_{o}^{F}\right),\\ a_{\mathrm{th}}^{⋆}\left(s_{t}^{G}\right) & \in \underset{a_{e}^{F}\in A_{e}^{G}\left(s_{t}^{G}\right)}{\mathrm{arg max}}V_{g}\left(s_{t}^{G},a_{e}^{F}\right). \end{array}$$

Here $\varepsilon_{\mathrm{BR}}=0.05$. Every evaluated ego candidate contributes the matched sample $\left({\tilde{o}}_{n},a_{g,n}^{F},v_{g,n},a_{\mathrm{th},n}^{⋆}\right)$ to $D_{g}$, where $v_{g,n}=V_{g}\left(s_{n}^{G},a_{g,n}^{F}\right)$.

For a nonempty finite follower bank with finite utilities and $\varepsilon_{\mathrm{BR}}\geq 0$, every utility maximizer belongs to $R_{o}^{\varepsilon }$, so the response set is nonempty. If either admissible candidate bank is empty, the teacher skips that state without generating action or value labels.

Before filtering, each role has 3×64=192 candidates. Thus one teacher state requires at most $192^{2}=36,864$ coupled previews, or 3,686,400 tactical rollout steps for $H_{G}=100$. At most 192 matched ego-action/value samples are generated per state. These are workload bounds; candidate filtering reduces the evaluated bank.

Before publication, the requested speed cap is screened against the speed-dependent tire envelope on the provisional corridor midpoint. For each trial $v\in V_{s}\left(V_{\mathrm{cap}}^{\mathrm{req}}\right)$, define the envelope-unclipped kinetic-energy template in Equation (13):

(13) $$\lambda_{k}=\frac{\sum_{j=0}^{k-1}\Delta \ell_{j}}{\sum_{j=0}^{N_{\mathrm{sta}}-2}\Delta \ell_{j}},\;{\hat{V}}_{k}\left(v\right)=\sqrt{V_{e}^{2}+\lambda_{k}\left(v^{2}-V_{e}^{2}\right)}.$$

Using the midpoint curvature ${\hat{\kappa }}_{k}$, the induced acceleration demand and peak envelope utilization are given by Equation (14):

(14) $$\begin{array}{cc} {\hat{a}}_{T,k}\left(v\right) & =\frac{{\hat{V}}_{k+1}^{2}\left(v\right)-{\hat{V}}_{k}^{2}\left(v\right)}{2\Delta \ell_{k}}+\frac{F_{\mathrm{drag}}\left({\hat{V}}_{k}\left(v\right)\right)}{m},\\ {\hat{a}}_{N,k}\left(v\right) & ={\hat{\kappa }}_{k}{\hat{V}}_{k}^{2}\left(v\right),\\ \hat{\rho }\left(v\right) & =\max_{0\leq k\leq N_{\mathrm{sta}}-2}\Phi_{\mathrm{tire}}\;\left({\hat{a}}_{T,k}\left(v\right),{\hat{a}}_{N,k}\left(v\right),{\hat{V}}_{k}\left(v\right)\right). \end{array}$$

The projection retains the largest trial cap within the envelope using Equation (15):

(15) $$\begin{array}{cc} V_{\mathrm{cap}}^{F}\left(a\right) & =\max\left\{v\in V_{s}\left(V_{\mathrm{cap}}^{\mathrm{req}}\right):\hat{\rho }\left(v\right)\leq 1\right\},\\ \Pi_{\mathrm{tire}}\left(a;\tilde{o}\right) & =a[V_{\mathrm{cap}}^{F}\left(a\right)]. \end{array}$$

Here $V_{e}$ is the current ego speed, $V_{s}$ is the uniformly sampled finite grid from $V_{\min}$ to $V_{\mathrm{cap}}^{\mathrm{req}}$, and a[v] changes only the cap component.

### 3.2. Game-Supervised Projected-Action TQC

The real-time policy maps the evolving duel geometry to the tactical feasible set at a fixed computational cost. Its 51-dimensional observation concatenates 27 duel-state features with two 12-dimensional track summaries centered at the ego and opponent. The duel features contain normalized Frenet states and accelerations, relative progress, lateral gap and closing rate, time to contact, closing-speed code, side lock, and a nine-state race-mode code. Each track summary reports mean curvature, peak absolute curvature, and minimum width over four consecutive bins. The vehicle-state and relative-motion features are constructed from GNSS/IMU-based ego-state estimates and LiDAR-based opponent-state estimates. The sensing pipeline and output perturbations are specified in Section 5.1.

The environment reward is defined in Equation (16):

(16) $$\begin{array}{cc} r_{t} & =w^{⊤}\phi_{t},\\ \phi_{t} & ={\left[\phi_{\mathrm{ot}},\phi_{\mathrm{ovb}},\phi_{\mathrm{col}},\phi_{\mathrm{lat}},\phi_{\mathrm{crush}},\phi_{\mathrm{app}},\phi_{\mathrm{sm}},\phi_{\mathrm{gs}},\phi_{\mathrm{sbs}},\phi_{\mathrm{wide}}\right]}^{⊤}. \end{array}$$

Appendix A gives the normalized definitions and weights. The same role-swapped features define Equation (10), aligning the teacher and online reward at the level of overtaking progress, clearance, smoothness, and usable corridor width.

Projected-action TQC. Truncated Quantile Critics (TQC) [34] discards the largest target atoms before the quantile-Huber backup. Environment replay D stores the final tactical action $a_{t}^{F}$. For a raw target-policy sample, the projected action and truncated target are given by Equation (17):   

(17) $$\begin{array}{cc} a_{t+1}^{F} & =\Pi_{\mathrm{tire}}\;\left(\Pi_{A}\left(a_{t+1}^{\mathrm{raw}}\right);{\tilde{o}}_{t+1}\right),\\ \ell_{t+1} & =\log\pi_{\theta }\left(a_{t+1}^{\mathrm{raw}}|{\tilde{o}}_{t+1}\right),\\ y_{t,k} & =r_{t}+\gamma \left(1-d_{t}\right)\left[z_{\left(k\right)}^{\prime }\left({\tilde{o}}_{t+1},a_{t+1}^{F}\right)-\alpha \ell_{t+1}\right],\\ Y_{t} & =\mathrm{sg}\;\left({\left\{y_{t,k}\right\}}_{k=1}^{K}\right),\;K<2N_{q}. \end{array}$$

Thus target critics and replay share the same projected-action support, while entropy remains the density of the raw stochastic policy. Actor and prior updates pass gradients through the non-smooth tire search using a straight-through (ST) projected-action surrogate in Equation (18):

(18) $$a^{F,\mathrm{ST}}=a^{B}+\mathrm{sg}\;\left[a^{F}-a^{B}\right].$$

This projected-action surrogate uses the exact projected action forward and an identity backward map through $\Pi_{\mathrm{tire}}$.

Two quantile heads share $h_{\zeta }=E_{\zeta }^{Q}\left(\tilde{o},a^{F}\right)$. For matched $\left({\tilde{o}}_{g},a_{g}^{F},v_{g},a_{\mathrm{th}}^{⋆}\right)∼D_{g}$, let $h_{\zeta ,g}=E_{\zeta }^{Q}\left({\tilde{o}}_{g},a_{g}^{F}\right)$; the updates are given by Equation (19):

(19) $$\begin{array}{cc} L_{\mathrm{critic}} & =J_{Q}+\lambda_{g}\;E_{D_{g}}\;\left[{\left(g_{\psi }\left(h_{\zeta ,g}\right)-N_{V}\left(v_{g}\right)\right)}^{2}\right],\\ L_{\mathrm{actor}} & =J_{\pi }+\lambda_{p}\;E_{D_{g}}\;\left[{∥N_{a}\left(\mu_{\theta }^{F}\right)-N_{a}\left(a_{\mathrm{th}}^{⋆}\right)∥}_{2}^{2}\right]. \end{array}$$

The matched samples in $D_{g}$ distill the teacher action and response-set value into observation-conditioned supervision over the sampled rollout contexts.

Here $J_{Q}$ and $J_{\pi }$ are the standard TQC critic and entropy-regularized actor losses; $N_{a}$ and $N_{V}$ normalize actions and teacher values. The actor encoder is independent, whereas the two quantile heads and the training-only robust-value head share the critic encoder. Consequently, D supplies Bellman transitions, whereas $D_{g}$ supplies matched teacher-action and response-set-value supervision. The action prior guides corridor placement, while the auxiliary value labels encourage the shared critic representation to retain response-set robustness information.

Writing the quantile-head-specific parameters as $\varphi_{1},\varphi_{2}$ and the auxiliary teacher-value loss as $L_{g}$, its gradient routes are given by Equation (20):

(20) $$\nabla_{\varphi_{1},\varphi_{2}}L_{g}=0,\;\nabla_{\zeta ,\psi }L_{g}\;\mathrm{are}\;\mathrm{generally}\;\mathrm{nonzero}.$$

Teacher-value supervision updates the shared encoder and can indirectly change quantile predictions. During critic optimization, only $J_{Q}$ updates the quantile-head-specific parameters. The action prior updates the independent actor.

**Figure 2:**
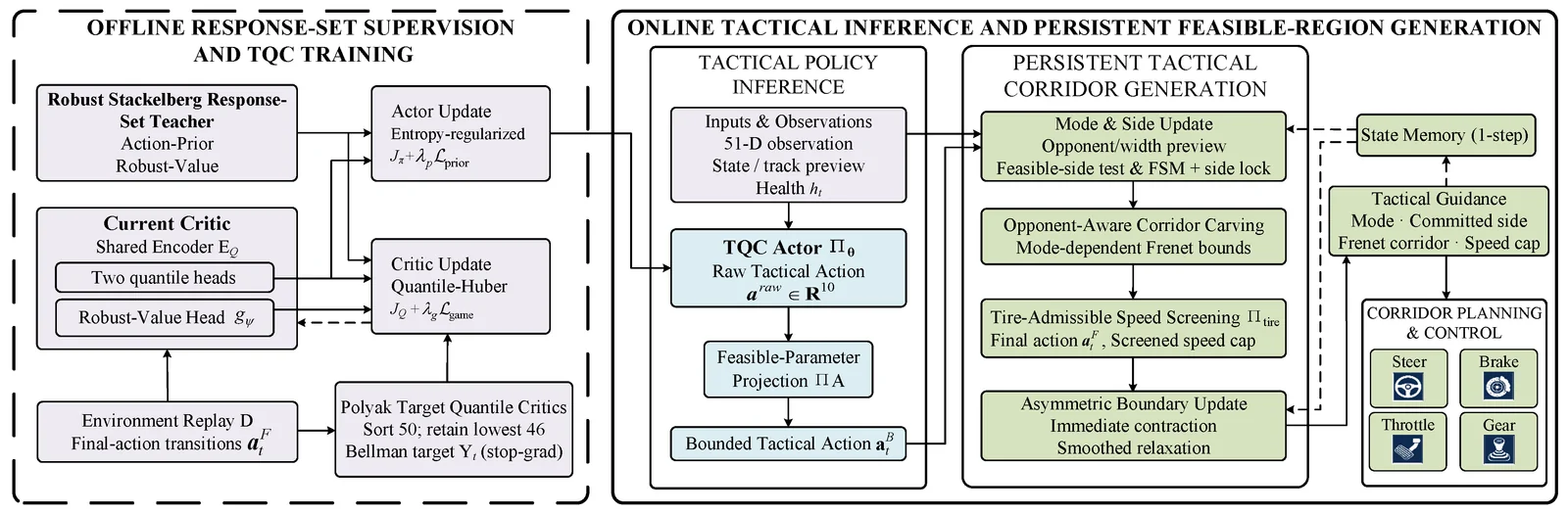
Overview of the proposed game-supervised tactical feasible-region framework.

Target-critic atoms are pooled and sorted, the lowest K=46 of the 50 atoms are retained, and Equation (17) supplies a stop-gradient Bellman target for the quantile-Huber loss. The actor uses the current critic quantiles in its entropy-regularized objective; target parameters are updated by Polyak averaging. Figure 2 summarizes the overall information flow.

### 3.3. Persistent Tactical Feasible-Region Interface

The state machine carries the selected passing opportunity across successive tactical updates. This temporal persistence complements the spatial corridor: the policy may refine its width and speed request as the encounter evolves. Let $\Delta s_{t}$ and $\Delta v_{t}$ be the sampled relative quantities defined in Equation (5). A feasible side is committed as the ego closes on the opponent, retained through Shadow and Overtake, and released when $P_{t}=1$ or upon an abort or execution-health failure. Health and abort take precedence over overtaking, body-clearance confirmation takes precedence over continued overtaking, and an unlocked ego enters Defend only when a rear opponent is closing.

The corridor carver uses a constant-Frenet-velocity opponent prediction initialized from LiDAR-based state estimates. At station $s_{k}$, the opponent position and footprint are evaluated at $t+{\hat{\tau }}_{k}$, where ${\hat{\tau }}_{k}=\left(s_{k}-s_{e}\right)/{\dot{s}}_{e}$ uses the current ego progress rate. For $B_{\mathrm{cap},k}=\left[\ell_{k},u_{k}\right]$, the vehicle footprint and one-sided exclusion are defined in Equation (21):

(21) $$\begin{array}{cc} \rho_{\mathrm{exc},k} & =d_{\mathrm{safe}}+\frac{1}{2}\sum_{j\in \{e,o\}}(L_{j}|\sin\xi_{j,k}|+W_{j}|\cos\xi_{j,k}\left|\right),\\ B_{\mathrm{cap},k} & =[\max\left\{n_{\mathrm{trk},k}^{-},-R^{\mathrm{cap}}\right\},\min\left\{n_{\mathrm{trk},k}^{+},L^{\mathrm{cap}}\right\}],\\ C_{k}^{\mathrm{side}}\left(\sigma ,\eta \right) & =\left\{\begin{array}{cc} {}[\ell_{k}+\eta {\left[n_{o,k}+\rho_{\mathrm{exc},k}-\ell_{k}\right]}_{+},u_{k}], & \sigma =+1,\\ \ell_{k},u_{k}-\eta {\left[u_{k}-n_{o,k}+\rho_{\mathrm{exc},k}\right]}_{+}], & \sigma =-1. \end{array}\right. \end{array}$$

**Figure 3:**
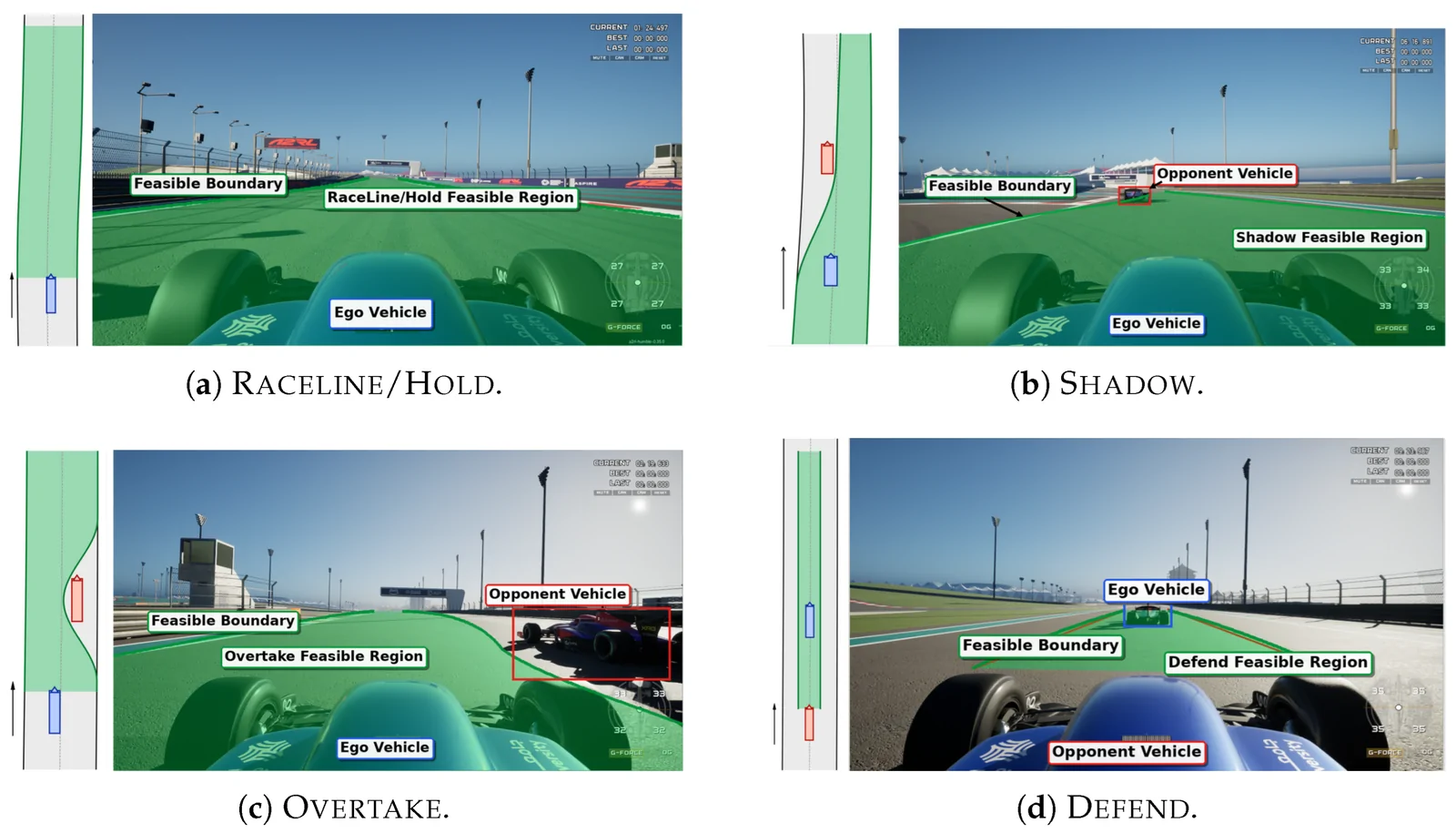
Illustrative geometry of the persistent tactical modes. Each panel pairs a schematic Frenet representation with the corresponding simulator view and visualizes how the mode shapes the nominal feasible region relative to the ego vehicle, opponent, and track boundaries. The simulator views were captured from the A2RL AutoVerse HIL platform described in Section 5.1.

Here σ=+1 and −1 select the left and right passing sides, respectively. With predicted longitudinal gap ${\hat{\Delta s}}_{k}$, Shadow uses $\eta =\mathrm{clip}(\left(g_{\mathrm{chase}}-{\hat{\Delta s}}_{k}\right)/\left(g_{\mathrm{chase}}-g_{\mathrm{ot}}\right),0,1)$, whereas Overtake uses full exclusion (η=1) on the locked side. Raceline uses the capped track interval; Defend intersects it with a width-$w_{\mathrm{safe}}$ interval centered at the clipped offset $n_{o,k}+b_{\mathrm{lat}}$; and Hold retains only the connected component containing the ego vehicle. Figure 3 illustrates these geometric semantics.

Algorithm 1 summarizes the complete route from offline interaction supervision to online corridor publication. Immediate contraction and smoothed relaxation allow the feasible region to respond quickly to reduced space while expanding progressively as the passing channel opens. The preceding corridor is revalidated for one update when a new interval loses the required width. Reuse does not renew the corridor’s age. A second consecutive width failure or failed revalidation releases the side lock and requests Hold/braking; passing resumes only after a new corridor is validated. Let $h_{t}$ denote the latest execution-health status available at tactical update *t*. The tactical publisher supplies $G_{t}=\left(m_{t},\sigma_{t}^{\mathrm{lock}},V_{\mathrm{cap},t}^{F},C_{t}\right)$ to the executor.

Together, the robust Stackelberg response-set teacher, projected-action TQC policy, and persistent tactical feasible-region interface transform opponent-aware interaction reasoning into side-consistent Frenet bounds and speed guidance for constrained motion planning. By adapting the corridor geometry while preserving the selected passing side, the framework maintains a consistent overtaking intention throughout the interaction.

## 4. Tactical Corridor Execution with Sequential SOCP and RC-NMPC

The execution layer uses Algorithm 2 to realize the persistent tactical feasible region as a continuous path–speed reference and actuator commands. The published corridor defines the spatial optimization domain, while the speed request sets the tactical pace throughout the maneuver.

For the $N=N_{\mathrm{sta}}$ spatial stations, let $z_{k}={\left[n_{k},\xi_{p,k}\right]}^{⊤}$ and $z_{T}={\left[n_{T},0\right]}^{⊤}$, where $n_{T}$ is the clipped raceline in Raceline and the terminal corridor midpoint in the other modes. The set $Z_{k}=\{{\left[n,\xi_{p}\right]}^{⊤}:{\underline{n}}_{k}\leq n\leq {\bar{n}}_{k},1-\kappa \left(s_{k}\right)n\geq \varepsilon_{s},\;|\xi_{p}\left|\;\leq \;\xi_{p,\max}\right\}$ combines the footprint-eroded corridor and Frenet-domain bounds. Sequential iteration *j* linearizes Equation (7) as ${\hat{f}}_{s,k}^{j}=A_{k}^{j}z_{k}+B_{k}^{j}\omega_{k}+b_{k}^{j}$ and solves Equation (22):

(22) $$\begin{array}{cc} y^{j+1}=\underset{y}{\mathrm{arg min}}\; & \sum_{k=0}^{N-1}w_{\omega }\sigma_{k}^{\omega }+w_{T}^{⊤}\epsilon_{T}\\ s.t.\; & z_{0}={\left[n_{e},\xi_{e}+\beta_{e}\right]}^{⊤},\\ \; & z_{k+1}-z_{k}=\frac{\Delta s_{\mathrm{sta}}}{2}\left({\hat{f}}_{s,k}^{j}+{\hat{f}}_{s,k+1}^{j}\right),\\ \; & z_{k}\in Z_{k},\;\left|\omega_{k}\right|\leq \omega_{\max},\\ \; & {∥{\left[2\omega_{k},\sigma_{k}^{\omega }-1\right]}^{⊤}∥}_{2}\leq \sigma_{k}^{\omega }+1,\\ \; & |z_{N-1}-z_{T}|\;⪯\;\epsilon_{T},\;\epsilon_{T}⪰0. \end{array}$$

Here y collects $z_{k}$, $\omega_{k}$, $\sigma_{k}^{\omega }$, and $\epsilon_{T}$. The terminal quantities are $\epsilon_{T}={\left[\epsilon_{n},\epsilon_{\xi_{p}}\right]}^{⊤}$ and $w_{T}={\left[w_{n,T},w_{\xi_{p},T}\right]}^{⊤}$, with $\omega_{\max}=\tan\delta_{\max}/\left(l_{f}+l_{r}\right)$. The dynamics apply for k=0,…,N−2 and the node constraints for k=0,…,N−1. The conic epigraph penalizes squared path curvature, while $\epsilon_{T}$ softens the terminal target. Each sequential update is re-evaluated with the nonlinear Frenet model.

The forward–backward sweep converts the tactical speed request into a tire-envelope-screened speed reference over the optimized path. Let $\Delta \ell_{k}={∥p_{k+1}-p_{k}∥}_{2}$ and $\kappa_{p,k}$ denote the segment length and path curvature. The envelope supplies the traction and braking limits $a_{T,k}^{+}$ and $b_{T,k}^{-}$ at $\left(\kappa_{p,k}V^{2},V\right)$, after subtracting the resistance acceleration in traction and adding it in braking. The steady tactical request is first converted into a braking-reachable stationwise cap $V_{\mathrm{cap},k}^{P}$. Intersecting it with the largest envelope-admissible curvature speed gives $V_{\lim,k}=\min\left\{{\bar{V}}_{\mathrm{curv},k},V_{\mathrm{cap},k}^{P}\right\}$. The forward pass starts from $V_{0}^{f}=V_{e}$; its terminal value initializes $V_{N-1}^{r}=\min\left\{V_{N-1}^{f},V_{\lim,N-1}\right\}$. The two recursions are given by Equation (23):   

(23) $$\begin{array}{cc} V_{k+1}^{f} & ={\mathrm{clip}}_{\left[V_{\min},V_{\lim,k+1}\right]}\sqrt{{\left(V_{k}^{f}\right)}^{2}+2\Delta \ell_{k}a_{T,k}^{+}\left(V_{k}^{f}\right)},\\ V_{k}^{r} & =\min\left\{V_{k}^{f},V_{\lim,k},\sqrt{{\left(V_{k+1}^{r}\right)}^{2}+2\Delta \ell_{k}b_{T,k+1}^{-}\left(V_{k+1}^{r}\right)}\right\}. \end{array}$$

Evaluated for k=0,…,N−2 and k=N−2,…,0, the forward and backward recursions impose acceleration and downstream braking reachability, respectively. Trapezoidal integration of $1/V_{k}^{r}$ provides the time stamps used to resample the path–speed reference on the controller grid. Reference acceptance requires $|V_{0}^{r}-V_{e}|\;\leq \;\varepsilon_{V}$ (numerical speed tolerance) and simultaneous footprint clearance using opponent predictions updated at these arrival times.

The RC-NMPC tracks the resampled path–speed reference on a fixed prediction grid in an ego-centered local frame. Its state and input are $\chi_{k}={\left[x_{k},y_{k},\theta_{k}\right]}^{⊤}$ and $\nu_{k}={\left[V_{k},\delta_{k}\right]}^{⊤}$, respectively, where $\theta_{k}$ is the vehicle heading in this frame. The discrete kinematic bicycle model is given by Equation (24):

(24) $$f_{\mathrm{kb}}\left(\chi_{k},\nu_{k}\right)=\chi_{k}+T_{c}\left[\begin{array}{c} V_{k}\cos\theta_{k}\\ V_{k}\sin\theta_{k}\\ V_{k}\tan\delta_{k}/\left(l_{f}+l_{r}\right) \end{array}\right].$$

Here $\nu_{k}^{r}={\left[V_{k}^{r},\delta_{k}^{r}\right]}^{⊤}$, with $\delta_{k}^{r}=\arctan\left(\left(l_{f}+l_{r}\right)\kappa_{p,k}^{r}\right)$. With $\theta_{k}^{r}$ denoting the planned-path tangent angle in the same frame, the quantities $e_{c,k}={\left(n_{k}^{r}\right)}^{⊤}\left(p_{k}-p_{k}^{r}\right)$, $e_{\psi ,k}=\mathrm{wrap}\left(\theta_{k}-\theta_{k}^{r}\right)$ define $e_{k}={\left[e_{c,k},e_{\psi ,k}\right]}^{⊤}$, and $Q=\mathrm{diag}(q_{c},q_{\psi })$. The set $U_{t,k}\left(\nu_{k-1}\right)$ collects $|\delta_{k}|\;\leq \;\delta_{\max}$, $|V_{k}-V_{k-1}|\;\leq \;\Delta V_{\max}$, and $|\delta_{k}-\delta_{k-1}|\;\leq \;{\dot{\delta }}_{\max}T_{c}$. Its speed component obeys $V_{\min}\;\leq \;V_{k}\;\leq \;\min\left\{v_{x,\max},V_{\mathrm{cap},t,k}^{C}\right\}$, where the stagewise cap is obtained by rate-limiting the tactical request from the previous speed command. Using ${∥x∥}_{M}^{2}=x^{⊤}Mx$, the controller solves Equation (25):

(25) $$\begin{array}{cc} \min_{\nu_{0:N_{c}-1}}\; & q_{\Delta \delta }{\left(\delta_{0}-\delta_{-1}\right)}^{2}+\sum_{k=0}^{N_{c}-1}\left(∥e_{k+1}{∥}_{Q}^{2}+{∥\nu_{k}-\nu_{k}^{r}∥}_{R_{\nu }}^{2}\right)\\ s.t.\; & \chi_{0}=0,\;\chi_{k+1}=f_{\mathrm{kb}}\left(\chi_{k},\nu_{k}\right),\\ \; & \nu_{k}\in U_{t,k}\left(\nu_{k-1}\right),\;k=0,\ldots ,N_{c}-1. \end{array}$$

Here $\nu_{-1}={\left[V_{-1},\delta_{-1}\right]}^{⊤}$ is the command applied in the preceding control cycle.
**Algorithm 2** Sequential SOCP and RC-NMPC execution.**Require:** tactical guidance $G_{t}$ or a braking-fallback request, current state, previous valid reference  1:set valid if $G_{t}$ is available  2:**if** valid **then**  3:    erode the track by the ego footprint, intersect it with $C_{t}$, and choose the mode-dependent terminal target  4:    initialize the spatial path from the previous valid solution or the corridor midpoint  5:    **for** each configured sequential-convexification iteration **do**  6:        solve SOCP (22) and update the linearization  7:    **end for**  8:    set valid if the path passes the solver and domain checks  9:**end if**10:**if** valid **then**11:    query $A_{\mathrm{tire}}\left(V\right)$ and construct $V_{\mathrm{cap},k}^{P}$ from $V_{\mathrm{cap}}^{F}$, measured speed, and available braking12:    intersect $V_{\mathrm{cap},k}^{P}$ with the curvature-speed envelope, run (23), and compute the reference arrival times13:    update the opponent prediction at these times; set valid only if initial-speed consistency, tire, and simultaneous footprint-clearance checks pass14:**end if**15:**if** valid **then**16:    time-resample the path–speed reference and rate-limit $V_{\mathrm{cap},t}^{F}$ to obtain $V_{\mathrm{cap},t,k}^{C}$17:    solve RC-NMPC (25) and update valid from its status18:**end if**19:**if** valid **then**20:    map $\left(V_{0|t}^{c⋆},\delta_{0|t}^{c⋆}\right)$ to throttle, brake, steering, and gear21:**else**22:    reuse an unused previous reference once only if current-state checks pass and no tactical braking is requested; otherwise request current-state Hold and issue rate-limited braking with the preceding steering angle23:**end if**24:**return** four-channel command and updated execution-health status

## 5. Experimental Results and Discussion

### 5.1. Experimental Platform and Configuration

**Figure 4:**
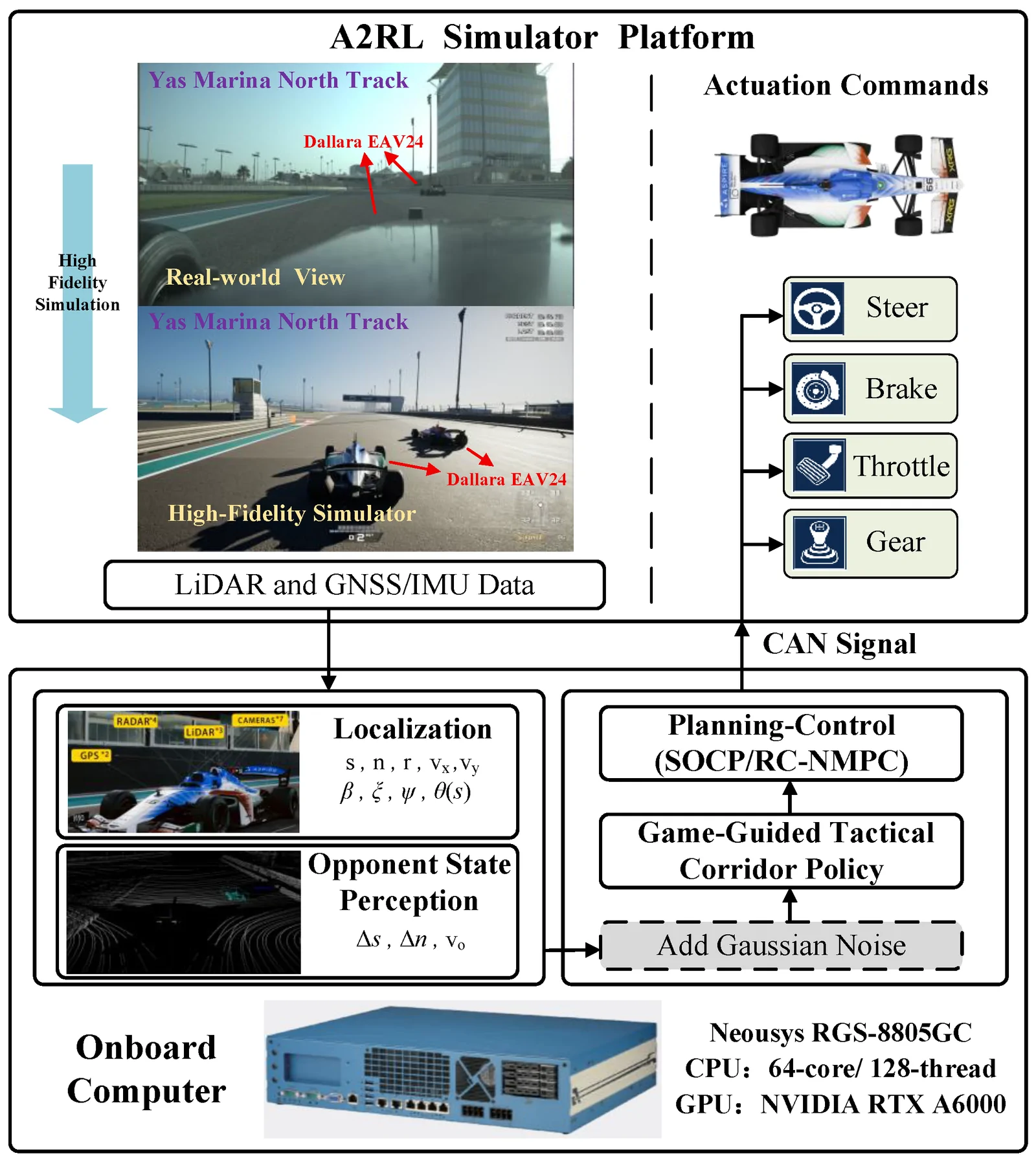
Hardware-in-the-loop architecture coupling the A2RL high-fidelity AutoVerse simulator with the RGS-8805GC onboard computer used in the full-scale race vehicle.

The Abu Dhabi Autonomous Racing League (A2RL) provides a full-scale head-to-head racing environment for EAV24 vehicles on Yas Marina North, together with the high-fidelity AutoVerse simulation platform. Using this platform, we established a hardware-in-the-loop (HIL) system that coupled the simulator with the Neousys RGS-8805GC onboard computer used on the EAV24 race car in the full-scale competition (Figure 4). Both policy training and the in-domain HIL evaluation used Yas Marina North. The AutoVerse simulator ran on a separate simulation server and generated LiDAR and GNSS/IMU data, which were transmitted to the onboard computer over a dedicated Ethernet link using DDSI–RTPS over UDP/IP. All five software modules were executed on the RGS-8805GC, which returned throttle, brake, steering, and gear commands to the simulated vehicle through a Controller Area Network (CAN).

The perception, localization, tactical, planning, and control modules operate at 20, 100, 200, 40, and 100 Hz, respectively. Nominal module rates and measured detection/publication rates are reported separately. The localization module fuses GNSS/IMU measurements to provide the ego vehicle’s position, velocity, attitude, and acceleration, while the perception module processes measurements from three LiDAR sensors to estimate the opponent’s position, velocity, and heading. A planar extended Kalman filter (EKF) fuses GNSS pose, inertial yaw rate and vehicle speed, with 100 Hz prediction and timestamped history updates; older observations are rejected. The LiDAR clouds undergo ego-frame alignment, bird’s-eye-view (BEV) rasterization, YOLO11-OBB detection, Hungarian association and Kalman tracking. State prediction, track-age checks and message timeouts handle asynchronous or missing inputs. Independent zero-mean Gaussian noise is added to the localization outputs with standard deviations of 0.05 m, 0.10m/s, 0.002 rad, and $0.10\;m/s^{2}$, respectively, and to the perception outputs with standard deviations of 0.10 m, 0.22m/s, and 0.003 rad, respectively. A 10% independent packet-drop rate is also applied to both output streams before they are supplied to the downstream tactical and planning modules. The tactical module implements the framework presented in Section 3, while the planning and control modules generate the path–speed reference and closed-loop vehicle commands, respectively.

Software timing with stationary vehicles and three 10 Hz LiDAR streams gave receipt-to-fused-output latency of 65.9±5.7 ms across five 60 s windows (window mean ± SD). YOLO computation averaged 16.61 ms; detection/publication rates were 9.66/39.99 Hz, with periodic outputs reusing observations. Five 20 s EKF windows gave pose/twist receipt-to-output delays of 7.23/7.22 ms. These latencies include processing and scheduling.

During policy training and the primary Monte Carlo HIL evaluations, the opponent followed the minimum-curvature racing line; Section 5.3 additionally evaluates two defensive opponent policies. The non-defending reference reflects A2RL’s restrictions on close-range defensive maneuvers, providing a repeatable overtaking benchmark. The same opponent policy applies on Imola and Suzuka; independently switching opponents were tested separately as described below.

Execution times were recorded throughout 1000 Monte Carlo closed-loop HIL laps of the proposed method. Table 1 reports the mean, P95, and P99 latencies; each P99 latency remained within its scheduled period. No solver failure was recorded.

Table 2, Table 3, Table 4, Table 5 and Table 6 report means over five seeded runs, each with 1000 Monte Carlo closed-loop laps per setting. Learned policies used independent training seeds; non-learning baselines used matched evaluation runs. In each lap, the opponent initial condition was randomized relative to the ego vehicle in longitudinal position, lateral position, and heading. Lead-acquisition rate (LAR) is the percentage of evaluation laps reaching the Lead event defined after Equation (5), with each lap counted once. Time to lead acquisition (TTLA) is the elapsed time from evaluation start to the first Lead event, averaged over laps acquiring the lead. The minimum gap is the smallest net clearance between the ego and opponent body envelopes over a lap. Mean time to collision (TTC) and contact/track-violation rates describe interaction safety, while the path-feasibility ratio (PFR) is the fraction of feasible planning updates.

Evaluations retained the original configuration, with parameters fixed beforehand and initial conditions and disturbance seeds paired across methods. Tests increased noise SD (×2 or ×4), packet loss (20% or 30%) or added delay (50, 100, or 200 ms), changing one factor at a time. The independent opponent selected either of two feasible paths with equal probability every 5 s of simulation time, retaining its nominal speed reference. The methods shared the same seeded command sequence; the opponent had no teacher or ego-policy access.

The five runs shared 1000 held-out starts and matched disturbance seeds across methods. Table 4 reports between-seed SDs for the full method and ablations. Table 2, Table 3, Table 4, Table 5 and Table 6 give descriptive Wilson intervals for 5000 pooled encounters, separately from training-seed variability. The sensing-stress, independent-opponent and initial-speed tests used 1000 encounters per condition.

Training used the original network and hyperparameters in Appendix A on the RGS-8805GC with an AMD EPYC 7003-series CPU and one NVIDIA RTX 6000 Ada GPU (48 GB). The teacher queried 1000 states, retaining 800 and producing 12,800 supervision records (10,240 training; 2560 validation, split by encounter). Data preparation, including teacher computation, took 34.8 h; 100,000 optimization updates required 33.3 min per seed. Table 3 reports decision-function timings.

### 5.2. Overtaking Performance Under Representative Track Conditions

**Figure 5:**
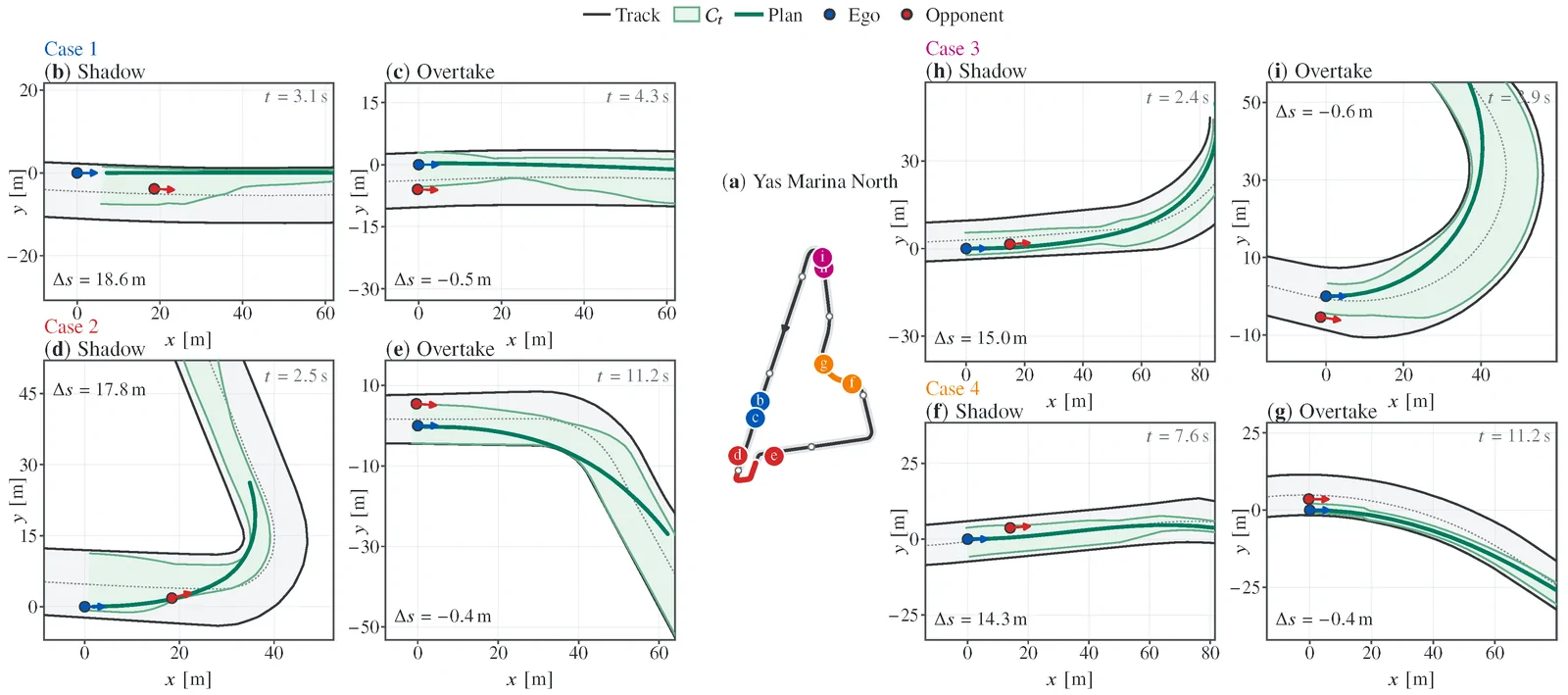
Representative overtaking situations on Yas Marina North.

To evaluate overtaking performance across different geometries and illustrate the evolution of the learned feasible region throughout each maneuver, Figure 5 presents four representative encounters at distinct locations on Yas Marina North. Panels (b)–(i) show the paired Shadow and Overtake spatial views at the locations marked in panel (a).

At the recorded starts of Cases 1–2, longitudinal gaps span 56.60–57.56 m, lateral offsets −3.12–3.32 m, heading differences −0.0037–0.0502 rad, and ego speed advantages 10.99–22.60 m/s.

Across the four encounters, the feasible region evolves from approach guidance into a committed passing channel. In Case 1, the Shadow corridor at Δs=18.6 m places the planned path on the selected side behind the opponent; at Δs=−0.5 m, the Overtake corridor excludes the opponent footprint and preserves a continuous path as the ego reaches the Lead event along the straight. Case 2 exhibits the same progression through a tight bend: the corridor first establishes the lateral approach at Δs=17.8 m and then follows the track curvature while maintaining the locked-side channel as the ego reaches the Lead event at Δs=−0.4 m. In Case 3, the corridor conforms to the sweeping corner during Shadow and carries the ego through the Lead event as the relative longitudinal gap decreases from 15.0 to −0.6 m. Case 4 selects the complementary side in the oppositely curved section; its gradual Shadow offset develops into a fully carved Overtake region as Δs decreases from 14.3 to −0.4 m, where the ego reaches the Lead event. These views demonstrate that the state-dependent corridor consistently converts side intention into a connected, track-conforming passing region across straight and curved sections. Cases 1 and 2 are examined in detail as representative high-speed overtakes on a straight and through a consecutive-corner sequence.

#### 5.2.1. Case 1:High-Speed Overtake on a Straight

**Figure 6:**
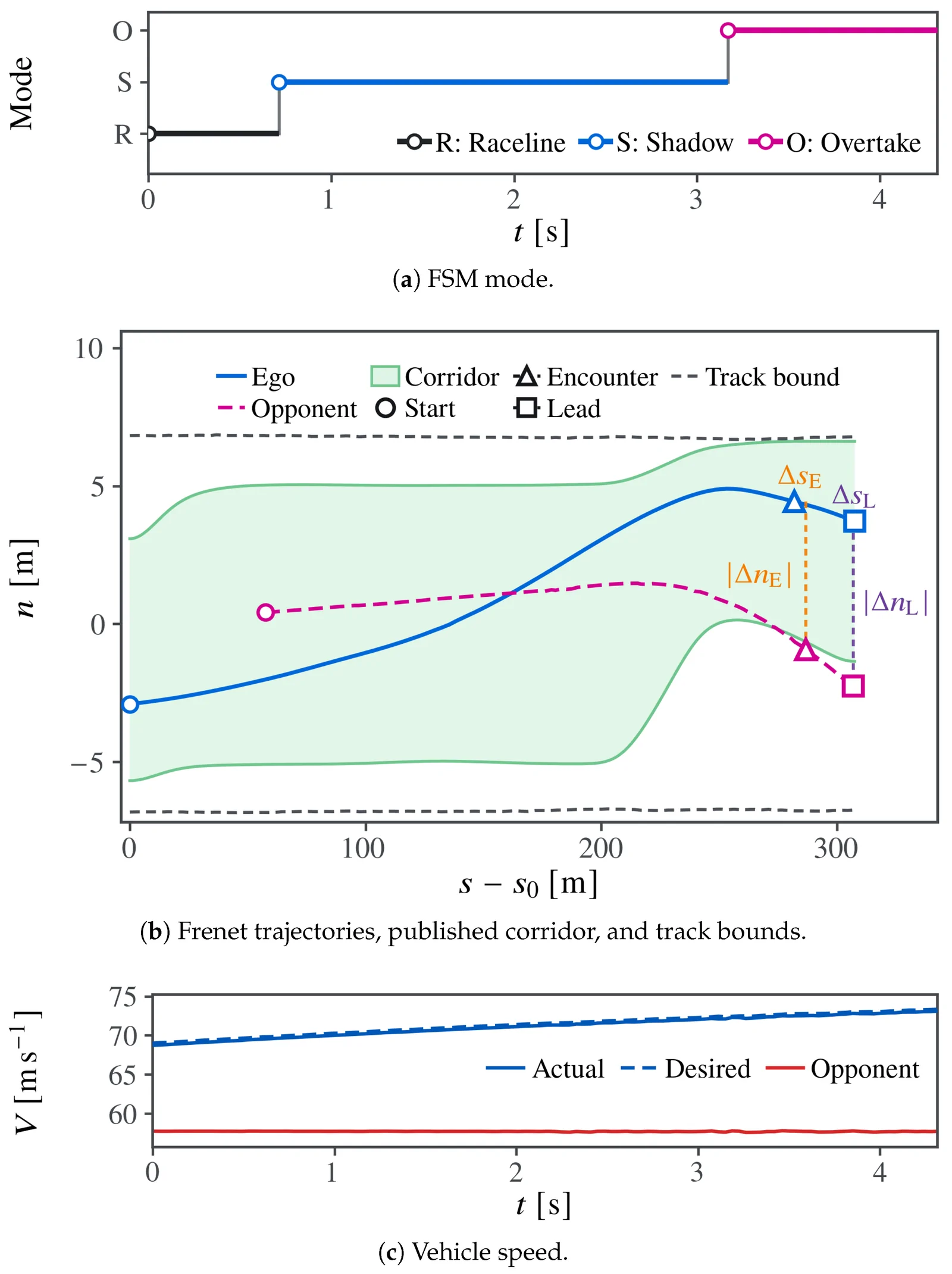
Tactical-mode sequence, Frenet trajectories and corridor, and speed response in Case 1.

Case 1 represents a high-speed overtake on the long straight. At the first recorded sample (t=0 in Figure 6), the opponent-minus-ego longitudinal, lateral and heading differences are 57.56 m, 3.32 m and −0.0037 rad; ego/opponent speeds are 68.73/57.74 m/s ($V_{e}-V_{o}=10.99$ m/s). In Figure 5, the Shadow view at Δs=18.6 m shows the corridor forming a side-consistent approach channel behind the opponent, with the planned path gradually displaced toward the selected passing side. When the relative longitudinal gap decreases to −0.5 m, the Overtake carver excludes the opponent-occupied region and retains a connected corridor as the ego reaches the Lead event.

Figure 6 summarizes the corresponding FSM sequence, Frenet trajectories, and speed response. In panel (b), $s-s_{0}$ denotes trackwise progress relative to the initial ego position, and *n* denotes the lateral position relative to the track centerline. The published feasible corridor remains within the physical track boundaries, and the actual ego trajectory stays inside this corridor throughout the overtaking maneuver. Triangles and squares mark the Encounter and Lead events, respectively. Encounter occurs at $\Delta s_{E}≃\left(L_{e}+L_{o}\right)/2$, when the longitudinal body envelopes first overlap after the corridor has established the lateral separation $|\Delta n_{E}|$. At the Lead marker, the lateral separation satisfies $|\;\Delta \;n_{L}|\geq \rho_{\mathrm{exc},L}$, and the footprint-exclusion condition in Equation (21) remains satisfied. The actual speed closely follows the desired profile and exceeds 70 m/s during lead acquisition.

**Figure 7:**
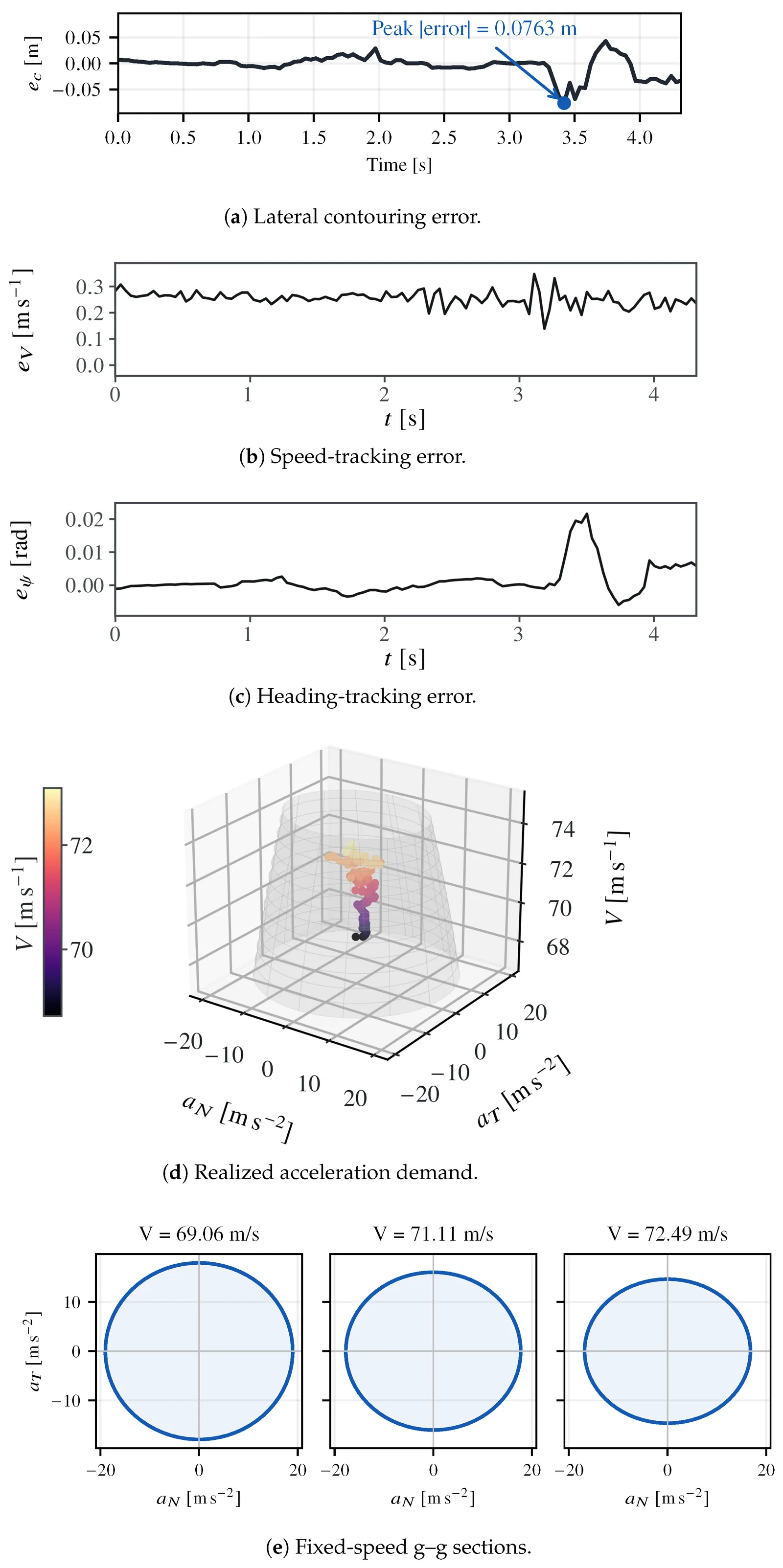
Closed-loop tracking errors and realized acceleration demand in Case 1.

Figure 7 presents the closed-loop realization of this passing channel. The peak absolute lateral contouring, speed-tracking, and heading errors are approximately 0.08 m, 0.35 m/s, and 0.025 rad, respectively. All recorded tangential–normal acceleration samples remain inside the speed-dependent tire envelope. The committed-side corridor is therefore realized as a trackable path–speed reference throughout the high-speed overtake.

#### 5.2.2. Case 2: High-Speed Overtake Through Consecutive Corners

**Figure 8:**
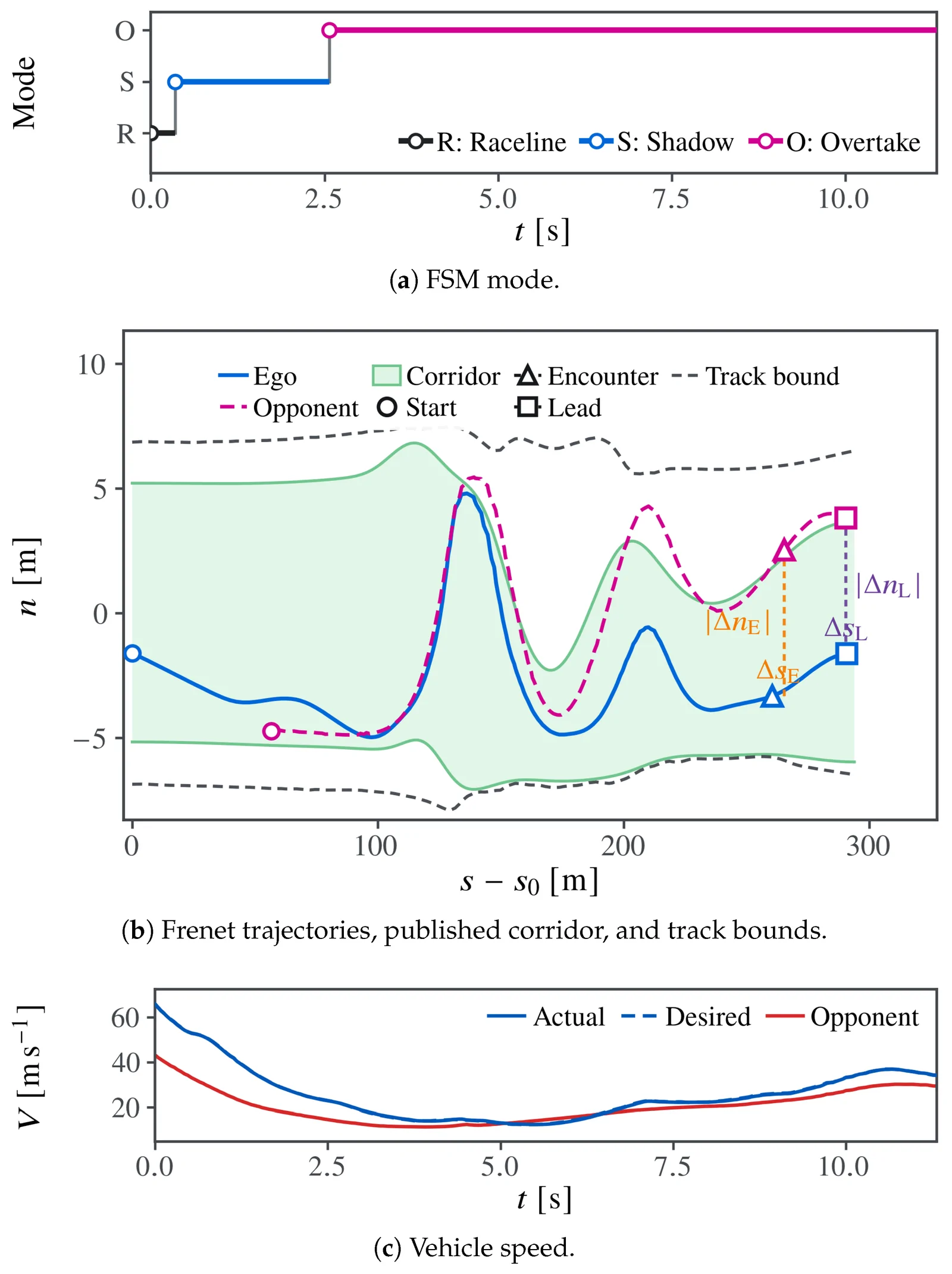
Tactical-mode sequence, Frenet trajectories and corridor, and speed response in Case 2.

Case 2 represents a high-speed overtake through successive curvature changes. At the first recorded sample (t=0 in Figure 8), the opponent-minus-ego longitudinal, lateral and heading differences are 56.60 m, −3.12 m and 0.0502 rad; ego/opponent speeds are 65.83/43.23 m/s ($V_{e}-V_{o}=22.60$ m/s). In Figure 5, the Shadow view at Δs=17.8 m shows the corridor following the approaching bend and guiding the planned path toward the selected lateral position. At Δs=−0.4 m, full Overtake exclusion preserves the same-side channel as the corridor guides the ego through the Lead event within the track boundaries.

Figure 8 summarizes the corresponding FSM sequence, Frenet trajectories, and speed response. The published feasible corridor follows the changing track boundaries, and the actual ego trajectory remains inside it while traversing the consecutive corners. At Encounter, the curved corridor has already established the lateral separation $|\Delta n_{E}|$ as the longitudinal body envelopes begin to overlap. At the Lead marker, $|\;\Delta \;n_{L}|\geq \rho_{\mathrm{exc},L}$, preserving lateral clearance throughout the overtaking maneuver. From an entry speed above 60 m/s, the actual speed closely follows the desired braking and re-acceleration profile throughout lead acquisition.

**Figure 9:**
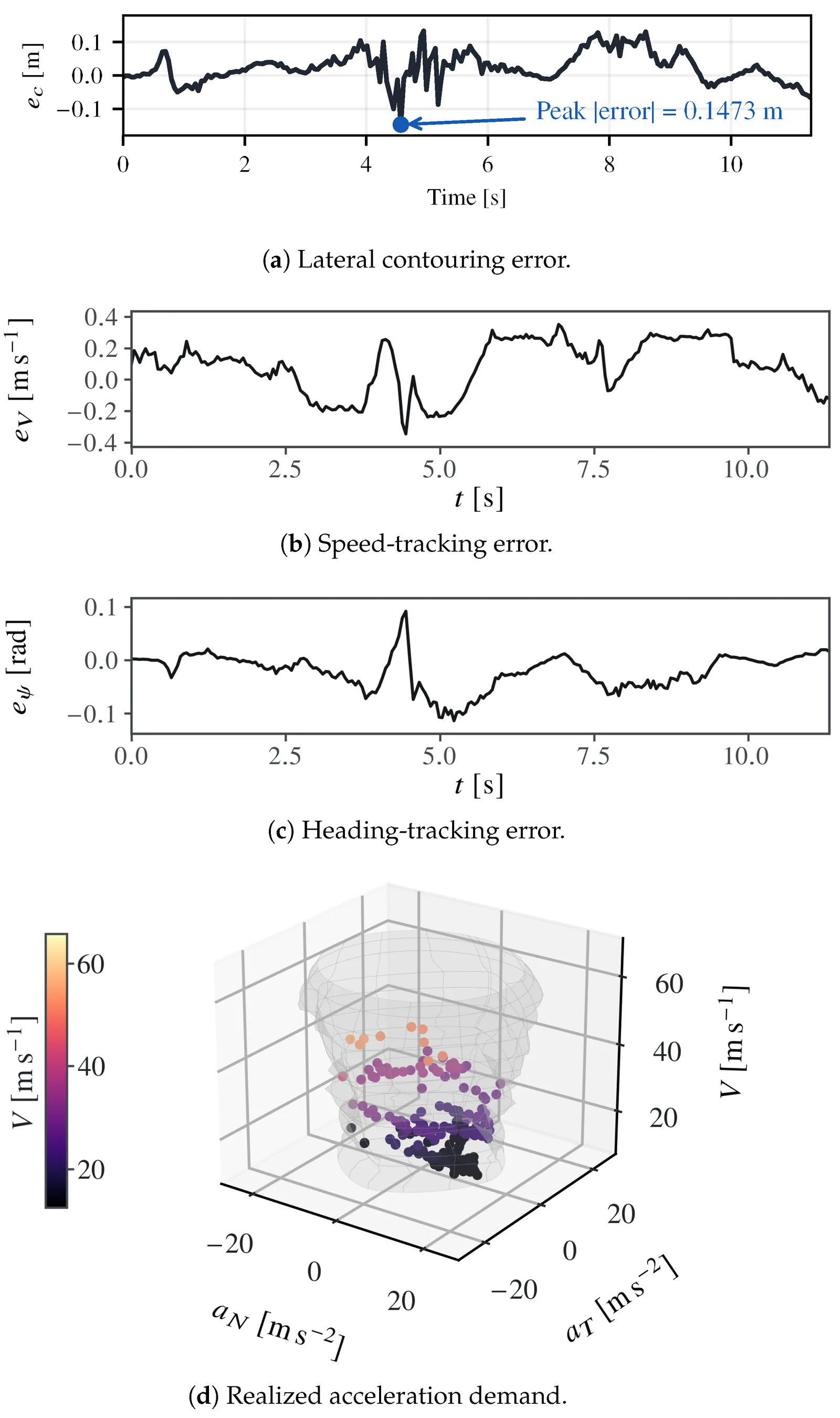

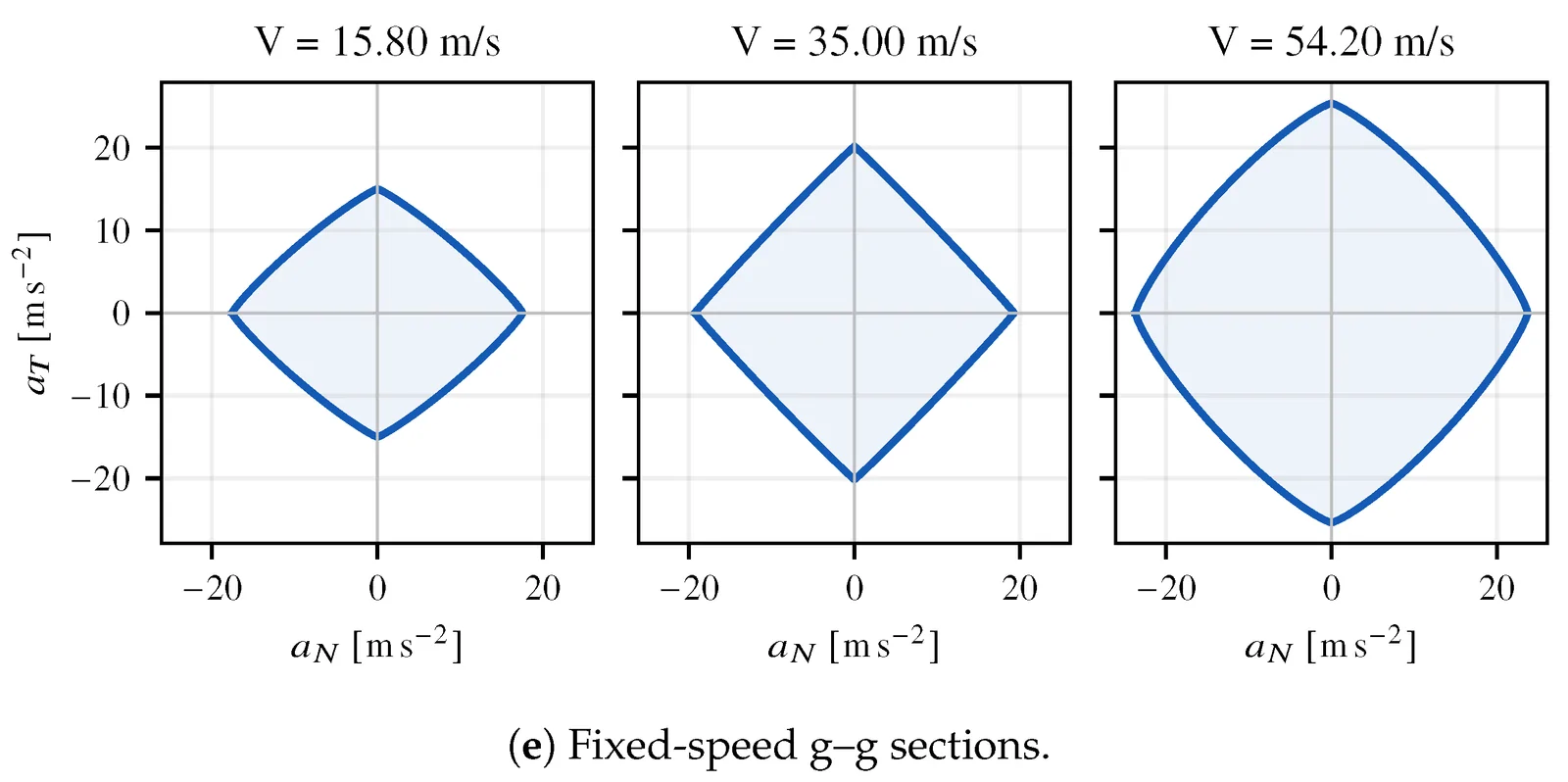
Closed-loop tracking errors and realized acceleration demand in Case 2.

Figure 9 presents the closed-loop realization of this curved passing channel. The peak absolute lateral contouring, speed-tracking, and heading errors are approximately 0.15 m, 0.35 m/s, and 0.11 rad, respectively, throughout the successive curvature and speed changes. All recorded tangential–normal acceleration samples remain inside the speed-dependent tire envelope. These results confirm accurate closed-loop realization of the side-locked corridor through consecutive corners with changing track width.

Across 1000 Monte Carlo laps per seeded run with the minimum-curvature opponent, the full method achieved an LAR of 94.7%, with contact and track-violation rates of 0.0%.

### 5.3. Adaptive Overtaking Against Defensive Opponent Policies

Two state-dependent defensive opponent policies were evaluated. The near-best-response defender evaluates the fixed admissible candidate bank at each response update and selects the retained near-best response least favorable to the ego, adapting its lateral position and speed as the duel evolves. The side-switching blocker moves into the ego’s committed passing channel and can execute one blocking-side switch, prompting the tactical publisher to exploit the complementary channel. Both policies satisfy the same track-boundary and tire-envelope constraints as the minimum-curvature opponent. The near-best-response defender shares the teacher candidate bank and utility and is therefore in-distribution. The robustness evaluation in Section 5.8 includes an independently seeded switching opponent.

**Figure 10:**
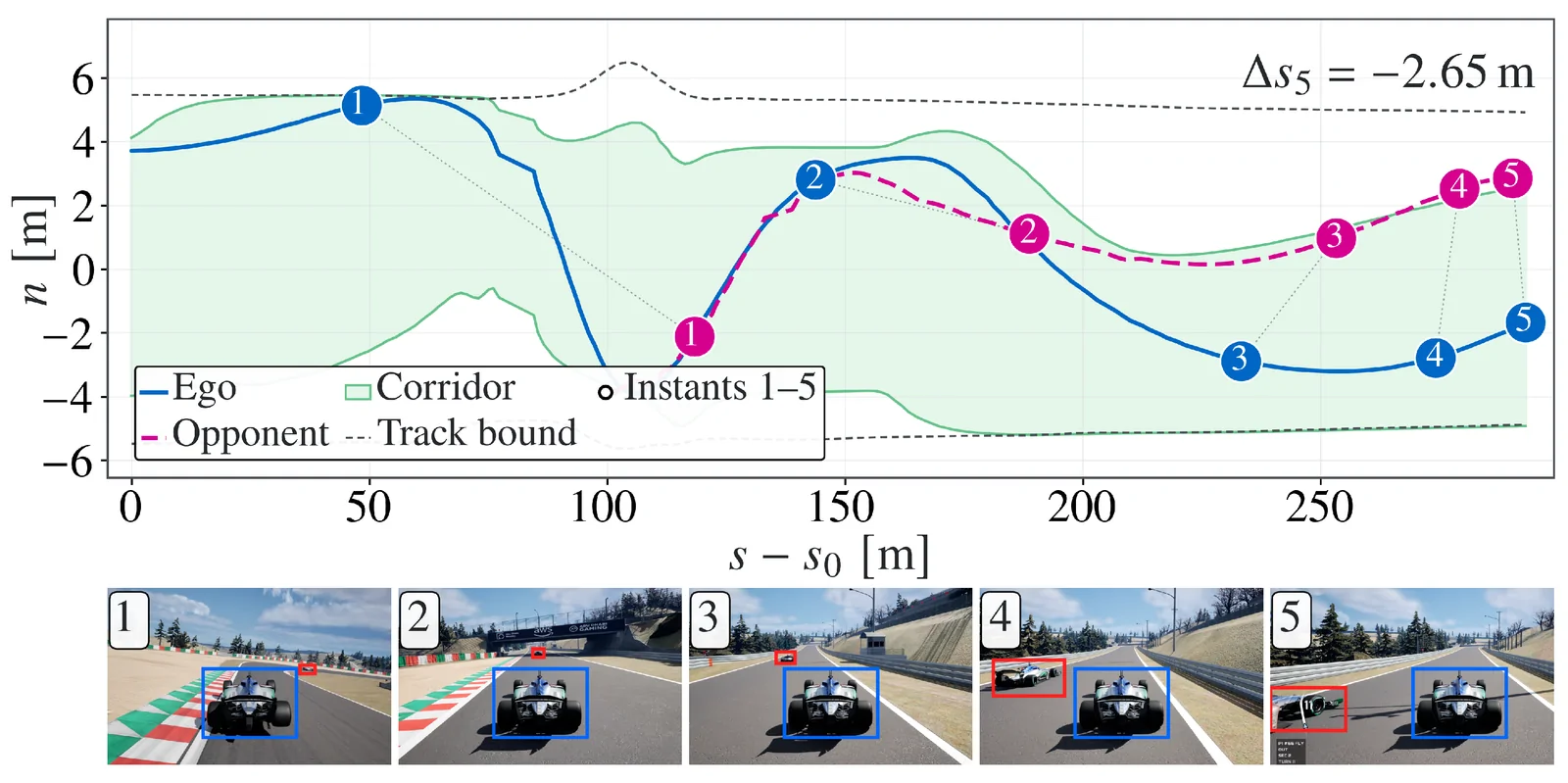
Closed-loop response to the side-switching blocker. Numbered markers correspond to the five illustrated states. Blue boxes denote the ego vehicle and red boxes denote the opponent.

Figure 10 presents a representative closed-loop ego response to the side-switching blocker at five instants. Across these instants, the opponent’s Frenet lateral coordinate spanned 5.15 m. Between instants 1 and 2, it crossed the track centerline from $n_{o}=-2.29$ to $n_{o}=1.10$ m, a 3.39 m shift into the ego’s initial passing channel. The tactical publisher responded by committing the complementary side and publishing its feasible corridor. The ego followed this corridor from $n_{e}=2.83$ m at instant 2 to $n_{e}=-2.89$ m at instant 3, completing a 5.72 m lateral transition. By instant 4, the relative longitudinal gap had decreased to $\Delta s_{4}=5.00$ m while the lateral separation reached $|\Delta n_{4}|=5.34$ m. By instant 5, the ego had acquired the lead at $\Delta s_{5}=-2.65$ m with $|\Delta n_{5}|=4.50$ m. The sequence shows that the learned publisher converts the open complementary channel into a continuous, track-conforming feasible corridor under active blocking conditions.

Paired 1000-lap Monte Carlo HIL evaluations per seeded run against both defensive policies are reported in Table 2, together with the minimum-curvature-opponent reference, using identical initial conditions and exogenous randomizations. The $\varepsilon_{\mathrm{BR}}=0$ policy retains $\lambda_{p}=\lambda_{g}=1$ but limits each teacher response set to the utility-maximizing response. With $\varepsilon_{\mathrm{BR}}=0.05$, the proposed policy achieved LARs of 92.3% and 91.0% against the near-best-response defender and side-switching blocker, respectively, while maintaining zero contacts and PFRs of 0.9985 and 0.9972. Relative to zero tolerance, the nominal response set increased LAR by 3.2 percentage points against each defender, increased the mean per-lap minimum gap by 0.72 and 0.75 m, and eliminated the 0.2% and 0.3% contact rates. PFR increased by 0.0045 and 0.0052, with TTLA increases among laps acquiring the lead of 0.09 and 0.16 s. These results demonstrate effective lead acquisition, clearance preservation, and planning feasibility against state-dependent defensive responses.

### 5.4. Comparison with Tactical Baselines

All methods share the same vehicle plant, race-control interface, corridor projection, path and speed planners, and control module; only the tactical guidance generator differs. Non-interactive model predictive control (NI-MPC) predicts the opponent independently of the ego plan and incorporates the predicted occupancy into its obstacle constraints [4,5]. Rule-based game-theoretic guidance (RB-GT) selects its tactical mode using a fixed-threshold state machine parameterized by $g_{\mathrm{chase}}=20\;m$, $g_{\mathrm{ot}}=8\;m$, and $g_{\mathrm{abort}}=35\;m$. Iterative linear-quadratic games (iLQGames) computes coupled local responses [13]. The outputs of all baselines are mapped to a common tactical interface comprising a passing-side command, a speed cap, and corridor parameters before entering the shared planning and control stack. This setup isolates the effect of tactical guidance on closed-loop overtaking performance. The comparison results are summarized in Table 3, where Prop. denotes the proposed method. Minimum gap is the mean per-lap minimum net clearance between the vehicle body envelopes, and PFR is the fraction of planning cycles with a feasible SOCP solution.

Decision-function timings in Table 3 use matched inputs on the same target computer, excluding shared observation processing, planning, control and transport. Table 1 reports full-callback timings.

Across 1000 Monte Carlo closed-loop HIL laps per method and seeded run, the proposed method achieved a lead-acquisition rate of 94.7% and a mean time to lead acquisition of 4.82 s. Compared with the three baselines, the lead-acquisition rate increased by 31.2–66.2 percentage points, and the mean time to lead acquisition decreased by 3.01–6.41 s. Among the baselines, iLQGames achieved the highest lead-acquisition rate of 63.5%, demonstrating the benefit of coupled interaction reasoning. The further improvement obtained by the proposed method demonstrates the advantage of expressing tactical reasoning as a persistent feasible region for closed-loop execution. Compared with iLQGames, the proposed method increased the mean minimum gap from 2.31 to 5.83 m and reduced the contact rate from 1.4% to zero. A PFR of 1.000 indicates that the common SOCP planner found a feasible solution at every planning cycle. An average of 53 laps per seeded run ended without reaching the Lead event.

### 5.5. Ablation Study

Eight ablation variants evaluate the contributions of response-set supervision, stateful side commitment, adaptive corridor–speed guidance, and distributional value learning while retaining the same downstream planning and control stack. All variants were independently trained and evaluated under matched conditions. Without action prior (No-AP) sets $\lambda_{p}=0$ while retaining action-conditioned robust-value supervision, whereas without robust-value supervision (No-RV) sets $\lambda_{g}=0$ while retaining the response-set-optimal action prior. Without game guidance (No-GG) removes both supervision signals. Without FSM (No-FSM) jointly removes the hysteretic mode transitions and side lock, allowing the passing-side command to follow the current policy output at each tactical update. Fixed corridor geometry (Fixed-C) replaces the learned lateral-cap, clearance, lateral-bias, and width outputs with the same nominal values used in Fixed-C/S, while retaining the learned speed request. Fixed speed guidance (Fixed-S) fixes only $V_{\mathrm{cap}}^{\mathrm{req}}$ while retaining the learned corridor geometry. Fixed corridor and speed guidance (Fixed-C/S) combines both interventions. The passing-side and transition outputs remain learned in all three fixed-guidance variants. The final variant replaces TQC with Soft Actor-Critic (SAC) in the same observation and action spaces, actor architecture, reward function, replay protocol, and training budget.

The full method achieved the best performance across all reported metrics, including an LAR of 94.7%, a mean TTLA of 4.82 s, a zero contact rate, and a PFR of 1.000 (Table 4). The targeted supervision ablations separate the contributions of the two teacher signals. Removing the action prior reduced LAR by 3.9 percentage points and increased TTLA by 0.88 s, whereas removing robust-value supervision reduced LAR by 2.4 percentage points and increased TTLA by 0.49 s. Their joint removal in No-GG produced a larger 9.3-point LAR reduction, together with a decrease in minimum gap from 5.83 to 2.86 m, supporting the complementary roles of action and value supervision.

The guidance ablations further distinguish spatial adaptation from speed adaptation. Fixing the corridor geometry reduced LAR by 13.3 percentage points, increased TTLA to 7.86 s, and decreased PFR to 0.970. Fixing only the speed request produced a smaller 5.1-point LAR reduction and increased TTLA to 6.12 s, while retaining a PFR of 0.994. Their joint removal in Fixed-C/S caused the largest degradation, reducing LAR to 76.1%, increasing TTLA to 8.94 s, and decreasing PFR to 0.961. These results show that adaptive corridor geometry provides the primary spatial support for feasible overtaking, while adaptive speed guidance contributes complementary pacing and efficiency.

Compared with No-FSM, the complete stateful mechanism increased LAR by 12.4 percentage points and reduced the contact rate from 1.2% to zero, demonstrating the combined contribution of hysteretic mode transitions and persistent side locking. Replacing TQC with SAC reduced LAR by 6.2 percentage points and increased TTLA by 0.71 s, supporting the contribution of distributional value learning in the same tactical and execution architecture.

### 5.6. Hyperparameter Sensitivity Analysis

The sensitivity analysis examines the response-set tolerance and increased supervision weights around the nominal setting, using $\varepsilon_{\mathrm{BR}}=0.05$ and $\lambda_{p}=\lambda_{g}=1$ as the reference configuration. Each setting was independently trained and evaluated over 1000 Monte Carlo closed-loop HIL laps per seed under the same experimental conditions. For each value of $\varepsilon_{\mathrm{BR}}$, the corresponding teacher labels were regenerated before training.

The nominal setting provided the strongest overall balance, achieving the highest LAR, a zero contact rate, and full planning feasibility (Table 5). Reducing $\varepsilon_{\mathrm{BR}}$ from 0.05 to 0.025 shortened TTLA from 4.82 to 4.71 s, while LAR decreased to 92.4% and the minimum gap decreased from 5.83 to 4.55 m. At zero tolerance, TTLA fell further to 4.60 s, while LAR, minimum gap, and PFR decreased to 90.1%, 4.08 m, and 0.9888, respectively, with a contact rate of 0.9%. Increasing $\varepsilon_{\mathrm{BR}}$ to 0.10 enlarged the minimum gap to 6.22 m, while TTLA increased to 5.70 s. These results show that $\varepsilon_{\mathrm{BR}}$ regulates the balance between overtaking efficiency and spatial clearance. Doubling either loss weight maintained LAR above 93% and PFR at or above 0.9968, demonstrating stable performance with increased supervision weights.

### 5.7. Evaluation on Unseen Circuits

The tactical policy trained on Yas Marina North was evaluated on the unseen Imola and Suzuka circuits without retraining or parameter retuning. All planning and control parameters were also kept unchanged.

**Figure A1:**
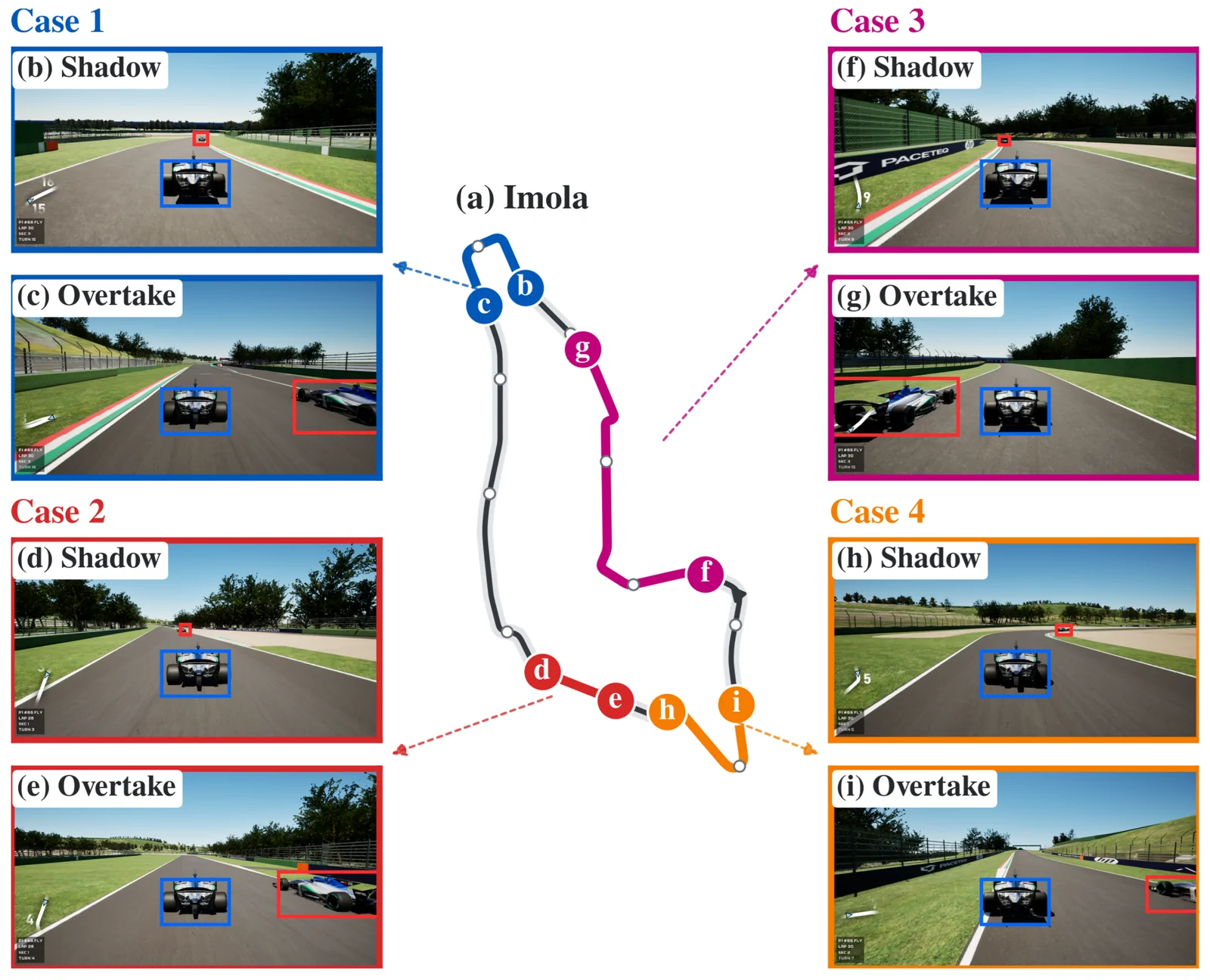
Representative overtaking events on the unseen Imola circuit. The central map localizes the Shadow and Overtake phases of four events, with the corresponding simulator views arranged around the map. Blue and red boxes indicate the ego and opponent vehicles.

**Figure A2:**
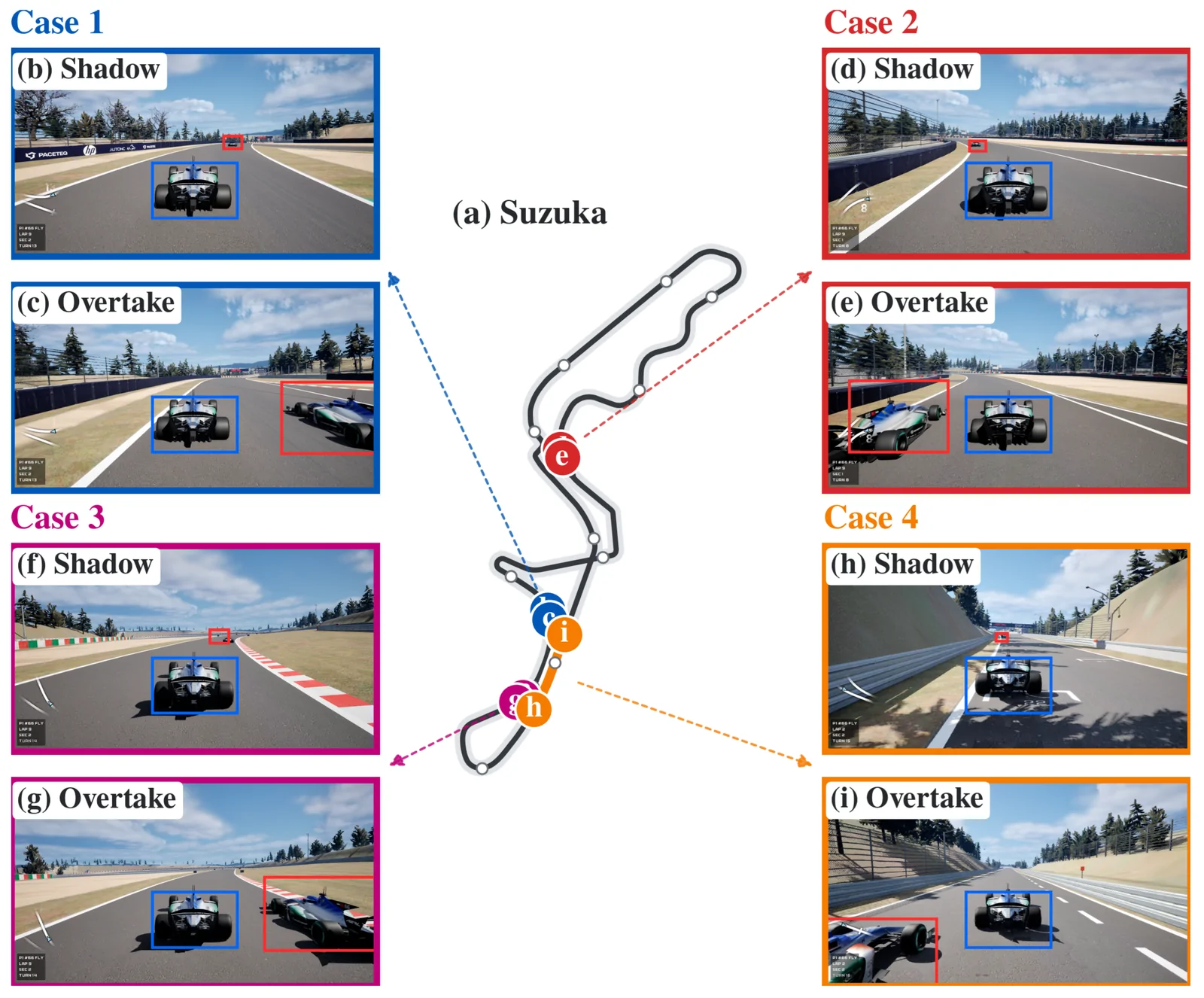
Representative overtaking events on the unseen Suzuka circuit. The central map localizes the Shadow and Overtake phases of four events across distinct local geometries. Blue and red boxes indicate the ego and opponent vehicles.

On both circuits, the opponent followed the minimum-curvature racing line. Figure A1 and Figure A2 in Appendix B each present four representative overtaking events. Every event pairs the Shadow and Overtake phases and localizes them on the corresponding track map. Imola features curved approaches and varied corner geometries, while Suzuka combines rapid direction changes, tight turns, and long acceleration sections. Together, these cases evaluate whether the learned tactical feasible-set representation sustains consistent overtaking decisions across new circuit geometries.

Across the eight illustrated events, the paired simulator views show the tactical state progressing from Shadow to Overtake on both straight and curved sections. The framework achieved LARs of 91.8% and 88.9% on Imola and Suzuka, respectively, while maintaining a zero contact rate on both circuits. The mean minimum gap remained above 5.0 m, and PFR remained above 0.993. The track-violation rates were 0.0% on Imola and 0.1% on Suzuka. These results demonstrate effective cross-circuit transfer of the learned tactical feasible-set representation.

### 5.8. Robustness and Initial-Speed Effects

LAR, TTLA and minimum gap follow Section 5.1. Table 7 compares the proposed method and iLQGames with sensing disturbances and an independent opponent.

The proposed method retains higher observed LAR and minimum gap, with lower contact rates, at every tested disturbance level. With an added delay of 200 ms, LAR is 81.6% versus 44.1%, and contact rates are 2.2% versus 6.9%. Against the independent opponent, LAR is 89.9% versus 56.2%.

With the Case 1 starting geometry and an initial opponent speed of 57.74 m/s, initial $V_{e}-V_{o}$ values of 1, 5 and 10 m/s yielded LARs of 78.9%, 87.5% and 90.2%, respectively (1000 trials per setting; 95% Wilson intervals: 76.26–81.32%, 85.31–89.41% and 88.20–91.89%). Disturbance seeds were paired; each trial lasted one circuit or ended earlier on contact, controller stopping or telemetry failure, with all attempts retained. Speeds evolved during interaction; acquiring the lead required accumulated relative track progress to exceed the initial gap.

## 6. Conclusions

This study developed a game-guided tactical-corridor framework for overtaking in autonomous racing. A TQC policy trained with robust Stackelberg response-set supervision takes sensor-derived opponent-state estimates as input and outputs passing-side intention, corridor parameters, and speed demand. Hysteretic side commitment and opponent-footprint carving convert these outputs into persistent Frenet corridors for constrained planning and closed-loop control. Monte Carlo closed-loop experiments on the EAV24 HIL platform demonstrated a 94.7% lead-acquisition rate and a mean time to lead acquisition of 4.82 s, with no collisions or track-boundary violations observed. The framework outperformed three matched baselines in lead-acquisition rate and time to lead acquisition and maintained lead-acquisition rates of 91.8% and 88.9% on the unseen Imola and Suzuka circuits, respectively. The original ablations support adaptive corridor shaping and persistent tactical state: the former preserves passing space, while hysteresis and side commitment maintain it across replanning. The response-set prior transfers interaction reasoning to the actor, while the shared critic representation learns its value without online candidate enumeration. In Section 5.8, the method retains higher observed LAR and minimum clearance, with lower contact rates than iLQGames, across the tested sensing disturbances and independent opponent.

A limitation of this study is that parameter selection has not been validated under conditions independent of the final evaluation. Future work will therefore separate tuning and evaluation conditions and extend testing to a full-scale race car. Track validation will progress from low-speed paired runs to high-speed overtakes, examining real-world sensing correlations, occlusion delays and tire/surface changes that may reduce passing clearance relative to HIL. This validation will also guide extension to multi-vehicle racing.

## Figures and Tables

**Table 1:** Execution-time statistics on the target computer.

Stage	Period [ms]	Mean [ms]	P95 [ms]	P99 [ms]
Tactical callback	5	1.213	1.895	2.486
Planning cycle	25	12.673	19.988	23.989
RC-NMPC	10	5.221	7.087	8.736

**Table 2:** Closed-loop performance against fixed and defensive opponents over 1000 Monte Carlo HIL laps per setting and seeded run. Brackets give 95% Wilson intervals for LAR and contact rates.

Metric	Minimum-Curvature Opponent		Near-Best Response Defender		Side-Switching Blocker	
$\varepsilon_{\mathrm{BR}}$	0.05	0	0.05	0	0.05	0
LAR [%] ↑	94.7 [94.04, 95.29]	90.1 [89.24, 90.90]	92.3 [91.53, 93.01]	89.1 [88.21, 89.93]	91.0 [90.17, 91.76]	87.8 [86.86, 88.68]
TTLA [s] ↓	4.82	4.60	5.13	5.04	5.41	5.25
Min. gap [m] ↑	5.83	4.08	5.19	4.47	4.91	4.16
Contact rate [%] ↓	0.0 [0.00, 0.08]	0.9 [0.67, 1.20]	0.0 [0.00, 0.08]	0.2 [0.11, 0.37]	0.0 [0.00, 0.08]	0.3 [0.18, 0.49]
PFR ↑	1.0000	0.9888	0.9985	0.9940	0.9972	0.9920

↑ indicates that higher values are preferable; ↓ indicates that lower values are preferable.

**Table 3:** Comparison over 1000 Monte Carlo closed-loop HIL laps per method and seeded run. Arrows indicate the preferred direction. Brackets give 95% Wilson intervals for LAR and contact rates. Decision times are measured separately over 500 calls per method.

Metric	Prop.	NI-MPC	RB-GT	iLQGames
LAR [%] ↑	94.7 [94.04, 95.29]	28.5 [27.27, 29.77]	51.6 [50.21, 52.98]	63.5 [62.16, 64.82]
TTLA [s] ↓	4.82	11.23	9.37	7.83
Min. gap [m] ↑	5.83	3.85	2.14	2.31
Contact rate [%] ↓	0.0 [0.00, 0.08]	0.7 [0.50, 0.97]	4.8 [4.24, 5.43]	1.4 [1.11, 1.76]
Mean TTC [s] ↑	8.52	6.92	5.64	5.73
Track violation [%] ↓	0.0	0.0	0.0	0.0
PFR ↑	1.000	0.998	0.987	0.995
Decision time, mean [ms] ↓	1.100	4.000	0.010	3.200
Decision time, P95 [ms] ↓	1.402	5.461	0.016	4.100
Decision time, P99 [ms] ↓	1.530	6.130	0.020	4.488

↑ indicates that higher values are preferable; ↓ indicates that lower values are preferable.

**Table 4:** Targeted and grouped component ablations in Monte Carlo closed-loop HIL experiments. Arrows indicate the preferred direction. Each variant uses five independent training seeds with 1000 laps per seed. Values following ± give between-seed SDs for LAR, TTLA, contact rate and minimum gap; brackets give descriptive 95% Wilson intervals for the pooled 5000 laps.

		Response-Set Supervision			State	Tactical Guidance			Critic
Metric	Full	No-AP ($\lambda_{p}=0$)	No-RV ($\lambda_{g}=0$)	No-GG	No-FSM	Fixed-C	Fixed-S	Fixed-C/S	SAC
LAR [%] ↑	94.7 ±0.25 [94.04, 95.29]	90.8 ±1.16 [89.97, 91.57]	92.3 ±0.94 [91.53, 93.01]	85.4 ±1.64 [84.39, 86.35]	82.3 ±1.10 [81.22, 83.33]	81.4 ±1.29 [80.30, 82.45]	89.6 ±1.20 [88.72, 90.42]	76.1 ±1.29 [74.90, 77.26]	88.5 ±0.94 [87.59, 89.35]
TTLA [s] ↓	4.82 ±1.44	5.70 ±1.68	5.31 ±1.60	6.38 ±1.93	6.71 ±2.01	7.86 ±2.33	6.12 ±1.89	8.94 ±2.62	5.53 ±1.66
Contact rate [%] ↓	0.0 ±0.00 [0.00, 0.08]	0.7 ±0.27 [0.50, 0.97]	0.5 ±0.26 [0.34, 0.74]	0.9 ±0.21 [0.67, 1.20]	1.2 ±0.22 [0.93, 1.54]	2.4 ±0.47 [2.01, 2.86]	0.6 ±0.19 [0.42, 0.86]	3.1 ±0.34 [2.65, 3.62]	0.5 ±0.14 [0.34, 0.74]
Min. gap [m] ↑	5.83 ±1.74	4.28 ±1.35	4.85 ±1.44	2.86 ±0.92	2.17 ±0.72	1.68 ±0.57	4.71 ±1.52	1.05 ±0.37	3.94 ±1.21
Mean TTC [s] ↑	8.52	6.35	7.02	4.93	4.31	3.52	6.79	2.86	6.17
PFR ↑	1.000	0.992	0.994	0.983	0.974	0.970	0.994	0.961	0.992

↑ indicates that higher values are preferable; ↓ indicates that lower values are preferable.

**Table 5:** Hyperparameter sensitivity over 1000 Monte Carlo closed-loop HIL laps per setting and seed. Arrows indicate the preferred direction. Brackets give 95% Wilson intervals for LAR and contact rates.

Setting	LAR [%] ↑	TTLA [s] ↓	Min. Gap [m] ↑	Contact Rate [%] ↓	PFR ↑
$\varepsilon_{\mathrm{BR}}=0.05$	94.7 [94.04, 95.29]	4.82	5.83	0.0 [0.00, 0.08]	1.0000
$\varepsilon_{\mathrm{BR}}=0.025$	92.4 [91.63, 93.10]	4.71	4.55	0.6 [0.42, 0.86]	0.9926
$\varepsilon_{\mathrm{BR}}=0$	90.1 [89.24, 90.90]	4.60	4.08	0.9 [0.67, 1.20]	0.9888
$\varepsilon_{\mathrm{BR}}=0.10$	93.0 [92.26, 93.67]	5.70	6.22	0.1 [0.04, 0.23]	0.9980
$\lambda_{p}=2$	93.1 [92.36, 93.77]	5.29	5.51	0.3 [0.18, 0.49]	0.9968
$\lambda_{g}=2$	93.4 [92.68, 94.06]	5.21	5.78	0.1 [0.04, 0.23]	0.9980

↑ indicates that higher values are preferable; ↓ indicates that lower values are preferable.

**Table 6:** Aggregate performance over 1000 Monte Carlo closed-loop HIL laps per unseen circuit and training seed. Brackets give 95% Wilson intervals for LAR and contact rates.

Metric	Imola	Suzuka
LAR [%]	91.8 [91.01, 92.53]	88.9 [88.00, 89.74]
TTLA [s]	5.19	4.08
Min. gap [m]	5.46	5.05
Mean TTC [s]	8.07	7.45
Contact rate [%]	0.0 [0.00, 0.08]	0.0 [0.00, 0.08]
Track-violation rate [%]	0.0	0.1
PFR	0.9968	0.9934

**Table 7:** Nominal, sensing-stress and independent-opponent results: 1000 encounters per row. TTLA uses lead-acquiring encounters; minimum gap uses all encounters. Continuous values are means ± encounter SD; brackets give 95% Wilson intervals.

Condition	Method	LAR [%] ↑ [95% CI]	TTLA [s] ↓	Min. Gap [m] ↑	Contact Rate [%] ↓ [95% CI]
Nominal	Prop.	94.7 [93.13, 95.93]	4.82 ± 1.44	5.83 ± 1.74	0.0 [0.00, 0.38]
	iLQGames	63.5 [60.47, 66.43]	7.83 ± 3.06	2.31 ± 0.91	1.4 [0.84, 2.34]
Noise SD ×2	Prop.	93.2 [91.47, 94.60]	5.07 ± 1.60	5.52 ± 1.70	0.1 [0.02, 0.56]
	iLQGames	61.4 [58.34, 64.37]	8.29 ± 3.39	2.17 ± 0.90	1.8 [1.14, 2.83]
Noise SD ×4	Prop.	90.1 [88.09, 91.80]	5.68 ± 1.66	4.97 ± 1.56	0.4 [0.16, 1.02]
	iLQGames	57.2 [54.11, 60.23]	9.14 ± 3.62	1.91 ± 0.85	2.9 [2.03, 4.13]
20% loss	Prop.	92.9 [91.14, 94.33]	5.16 ± 1.59	5.46 ± 1.62	0.1 [0.02, 0.56]
	iLQGames	60.6 [57.54, 63.58]	8.46 ± 3.60	2.11 ± 0.89	2.0 [1.30, 3.07]
30% loss	Prop.	89.4 [87.34, 91.16]	5.94 ± 1.77	4.83 ± 1.47	0.6 [0.28, 1.30]
	iLQGames	55.7 [52.61, 58.75]	9.71 ± 3.85	1.82 ± 0.80	3.5 [2.53, 4.83]
+50 ms	Prop.	92.5 [90.70, 93.97]	5.24 ± 1.59	5.39 ± 1.66	0.2 [0.05, 0.73]
	iLQGames	59.8 [56.73, 62.80]	8.61 ± 3.60	2.05 ± 0.87	2.2 [1.46, 3.31]
+100 ms	Prop.	88.7 [86.59, 90.52]	6.18 ± 1.99	4.68 ± 1.48	0.8 [0.41, 1.57]
	iLQGames	53.6 [50.50, 56.67]	10.25 ± 4.31	1.67 ± 0.77	4.3 [3.21, 5.74]
+200 ms	Prop.	81.6 [79.08, 83.88]	7.92 ± 2.37	3.71 ± 1.26	2.2 [1.46, 3.31]
	iLQGames	44.1 [41.05, 47.19]	12.88 ± 5.25	1.21 ± 0.60	6.9 [5.49, 8.64]
Independent opponent	Prop.	89.9 [87.88, 91.62]	5.82 ± 1.74	4.92 ± 1.52	0.3 [0.10, 0.88]
	iLQGames	56.2 [53.11, 59.25]	9.69 ± 3.77	1.88 ± 0.84	3.2 [2.28, 4.48]

↑ indicates that higher values are preferable; ↓ indicates that lower values are preferable.

## Data Availability

The experimental results data generated in this study are openly available on GitHub at https://github.com/fpocheese/overtaking-figure-data-and-video (accessed on 16 October 2026).

## Appendix A. Algorithm and Evaluation Parameters

Table A1 reports the principal numerical settings used in the study; the normalized reward features are defined below.

**Table A1:** Principal model, tactical-policy, training, and closed-loop settings. Symbols follow Equations (8) and (16).

Component	Parameter	Value
Vehicle geometry	*m*; $I_{z}$; $l_{f},l_{r}$	742 kg; 1500 kg m^2^; 1.75,1.35 m
Tire curves	$F_{z,0}$; $C_{x},C_{y}$	2079.72 N; 1.6144,1.5998
Tire curves	$\mu_{x},\mu_{y}$; $s_{x},s_{y}$; $q_{x,\mathrm{pk}},q_{y,\mathrm{pk}}$	2.2345,1.9745; −0.2555,−0.4787; 0.1296,0.140
Time scales	tactical decision step $T_{\mathrm{tac}}$; $T_{c}$; station spacing	0.005 s; 0.01 s; 2 m
Tactical bounds	$L^{\mathrm{cap}},R^{\mathrm{cap}}$; $d_{\mathrm{safe}}$; $V_{\mathrm{cap}}^{\mathrm{req}}$	[1.5,8.0] m; [0.2,0.8] m; [15,90] m s^−1^
Gap bounds	$g_{\mathrm{chase}}$; $g_{\mathrm{ot}}$; $g_{\mathrm{abort}}$	[18,24] m; [8,14] m; [28,45] m
Mode bounds	$b_{\mathrm{lat}}$; $q_{\mathrm{side}}$	[−1.5,1.5] m; [−1,1]
Response-set teacher	Sobol candidates per side code; $\varepsilon_{\mathrm{BR}}$; rollout steps $H_{G}$; $\gamma_{g}$	64; 0.05; 100; 0.9995
Projection and FSM	guard separation $\Delta_{g}$; clearance-confirmation count $N_{\mathrm{post}}$; stale-reference limit	0.5 m; 3 tactical updates; 1 cycle
TQC	critics; $N_{q}$; retained *K*; γ	2; 25; 46; 0.9995
TQC guidance	$\lambda_{p}$; $\lambda_{g}$; prior metric	1; 1; identity after action normalization
Training	learning rate; replay capacity; batch size; target-update coefficient	$3\times 10^{-4}$; $10^{5}$; 256; $5\times 10^{-3}$
Policy networks	actor encoder; critic outputs; auxiliary head	51→256→256; two quantile heads; training only
Reward	w, ordered as in Equation (16)	(30, −20, −100, −2, −5, 3, −0.1, −0.5, 0.5, 0.25)
Corridor	minimum width $L_{\min}^{\mathrm{corr}}$	3 m
Frenet health	$\varepsilon_{s}$; $\xi_{p,\max}$	0.05; 1.30 rad
Path health	sequential-convexification iterations	5
Speed profile	tire-cap grid intervals $H_{s}$; $V_{\min},v_{x,\max}$	150; 10,90 m s^−1^
RC-NMPC	steps $N_{c}$; $\delta_{\max}$; ${\dot{\delta }}_{\max}$; $\Delta V_{\max}$	15; 0.35 rad; 1.0 rad s^−1^; 0.5 m s^−1^
RC-NMPC weights	$q_{\Delta \delta },q_{c},q_{\psi }$; $R_{\nu }$	2,20,10; diag(1,0.5)

Here $n_{\mathrm{rl}}$ is the raceline offset at $s_{e}$, $W_{\mathrm{trk},k}=n_{\mathrm{trk},k}^{+}-n_{\mathrm{trk},k}^{-}$, $W_{\mathrm{trk}}=W_{\mathrm{trk},0}$, and $\rho_{\mathrm{exc}}=\rho_{\mathrm{exc},0}$. The normalized reward features are given by Equation (A1):

(A1) $$\begin{array}{cc} \phi_{\mathrm{ot}} & =⊮[m_{t}=OVERTAKE,m_{t+1}=RACELINE,P_{t}],\\ \phi_{\mathrm{ovb}} & =\phi_{\mathrm{ot}}\;\mathrm{with}\;\mathrm{roles}\;\mathrm{exchanged},\\ \phi_{\mathrm{col}} & =⊮[\mathrm{contact}\vee \mathrm{offtrack}],\\ \phi_{\mathrm{lat}} & =⊮\left[m_{t}=RACELINE\right]\mathrm{clip}\;{\left(\frac{n_{e}-n_{\mathrm{rl}}}{W_{\mathrm{trk}}/2},-1,1\right)}^{2},\\ \phi_{\mathrm{crush}} & =⊮[|\Delta s_{t}\left|<g_{\mathrm{ot}}\right]{\left(\frac{[\rho_{\mathrm{exc}}-{\left|\Delta n|\right]}_{+}}{\rho_{\mathrm{exc}}}\right)}^{2},\\ \phi_{\mathrm{app}} & =⊮\left[0<\Delta s_{t}<g_{\mathrm{chase}}\right]\mathrm{clip}\;\left(\frac{\Delta s_{t-1}-\Delta s_{t}}{g_{\mathrm{chase}}},0,1\right),\\ \phi_{\mathrm{sm}} & =\min\;\left\{1,\frac{∥N_{a}\left(a_{t}^{F}\right)-N_{a}\left(a_{t-1}^{F}\right){∥}_{2}^{2}}{10}\right\},\\ \phi_{\mathrm{gs}} & =\min\;\left\{1,\frac{1}{2N_{\mathrm{sta}}}\sum_{k}\sum_{\pm }{\left(\frac{n_{k,t}^{\pm }-n_{k,t-1}^{\pm }}{W_{\mathrm{trk},k}}\right)}^{2}\right\},\\ \phi_{\mathrm{sbs}} & =⊮[|\Delta s_{t}|<g_{\mathrm{ot}},|\Delta n|\geq \rho_{\mathrm{exc}}],\\ \phi_{\mathrm{wide}} & =\frac{1}{N_{\mathrm{sta}}}\sum_{k}\mathrm{clip}\;\left(\frac{n_{k,t}^{+}-n_{k,t}^{-}-L_{\min}^{\mathrm{corr}}}{W_{\mathrm{trk},k}-L_{\min}^{\mathrm{corr}}},0,1\right). \end{array}$$
